# Actinomycetes as Producers of Biologically Active Terpenoids: Current Trends and Patents

**DOI:** 10.3390/ph16060872

**Published:** 2023-06-12

**Authors:** Ekaterina V. Tarasova, Natalia A. Luchnikova, Victoria V. Grishko, Irina B. Ivshina

**Affiliations:** 1 Perm Federal Research Center, Ural Branch of the Russian Academy of Sciences, 13A Lenina Str., 614990 Perm, Russia; luchnikova.n@mail.ru (N.A.L.); grishvic@gmail.com (V.V.G.); ivshina@iegm.ru (I.B.I.); 2 Department of Microbiology and Immunology, Perm State University, 15 Bukirev Str., 614990 Perm, Russia

**Keywords:** terpenes, terpenoids, meroterpenoids, actinomycetes, biosynthesis, terpene synthase, biologically active compounds, genome mining

## Abstract

Terpenes and their derivatives (terpenoids and meroterpenoids, in particular) constitute the largest class of natural compounds, which have valuable biological activities and are promising therapeutic agents. The present review assesses the biosynthetic capabilities of actinomycetes to produce various terpene derivatives; reports the main methodological approaches to searching for new terpenes and their derivatives; identifies the most active terpene producers among actinomycetes; and describes the chemical diversity and biological properties of the obtained compounds. Among terpene derivatives isolated from actinomycetes, compounds with pronounced antifungal, antiviral, antitumor, anti-inflammatory, and other effects were determined. Actinomycete-produced terpenoids and meroterpenoids with high antimicrobial activity are of interest as a source of novel antibiotics effective against drug-resistant pathogenic bacteria. Most of the discovered terpene derivatives are produced by the genus *Streptomyces*; however, recent publications have reported terpene biosynthesis by members of the genera *Actinomadura*, *Allokutzneria*, *Amycolatopsis*, *Kitasatosporia*, *Micromonospora*, *Nocardiopsis*, *Salinispora*, *Verrucosispora*, etc. It should be noted that the use of genetically modified actinomycetes is an effective tool for studying and regulating terpenes, as well as increasing productivity of terpene biosynthesis in comparison with native producers. The review includes research articles on terpene biosynthesis by *Actinomycetes* between 2000 and 2022, and a patent analysis in this area shows current trends and actual research directions in this field.

## 1. Introduction

Terpenes and their *O*-containing derivatives (terpenoids) are the largest (more than 80,000 compounds) and structurally most diverse group of secondary metabolites derived from natural sources. Based on the number of isoprene units, terpene derivatives are classified into mono- (C10), sesqui- (C15), di- (C20), sester- (C25), tri- (C30), sesquar- (C35), and tetra- (C40) terpenes. Terpene derivatives are widely used in the food, cosmetics, and fragrance industries [1]. They exhibit various biological activities (antitumor, anti-inflammatory, antimicrobial, antiviral, immunomodulatory, antioxidant, antifungal, etc.) and are promising therapeutic agents [2]. Production of terpene derivatives from natural sources (plants, fungi, and marine organisms) does not meet industrial needs, while chemical synthesis is often a multi-stage and low selective process.

In the last 15–20 years, it has become obvious that bacteria also produce terpenes and terpenoids and that most of the produced metabolites are represented by new compounds. Currently, the search for microorganisms synthesizing terpene derivatives is underway and microbial biosynthetic platforms are developed using such microorganisms [3]. Microbial biosynthesis has advantages over traditional methods of obtaining terpenoids: a short life cycle of microorganisms, which reduces the production time of compounds to several days, high productivity throughout the fermentation process, and the use of cheap renewable resources to produce target products [4]. The ability for terpene biosynthesis has been described for actino-, proteo-, and cyanobacteria [5,6,7].

Actinomycetes are one of the largest, most diverse and well-studied group of bacteria represented by the genera such as *Mycobacterium*, *Nocardia*, *Rhodococcus*, *Streptomyces*, *Arthrobacter*, *Actinomyces*, *Corynebacterium*, *Micrococcus*, *Frankia*, *Micromonospora*. They are characterized by a wide range of genetic, morphological, and physiological characteristics, as well as metabolic capabilities [8]. Actinomycetes are well-known producers of secondary metabolites (polyketides, antibiotics, siderophores, biosurfactants, etc.) and enzymes (amylase, lipase, cellulase, protease), which can be used in pharmaceutical, agricultural, food, pulp and paper, and other industries [9,10,11,12,13,14,15,16,17,18,19]. Of 23,000 bioactive microbial metabolites, about 10,000 metabolites were isolated from actinomycetes [15], among which compounds with herbicidal [20], antitumor [21], antifungal [22], immunomodulating [23,24,25], and other activities were found. Most of the known antimicrobials (streptomycin, streptothricin, actinomycin, etc.) were originally produced by actinomycetes, especially by the genus *Streptomyces* [26]. Secondary metabolites of actinomycetes are widely used in various human activities and their use will rise in the future (Table 1).

**Figure 1:**
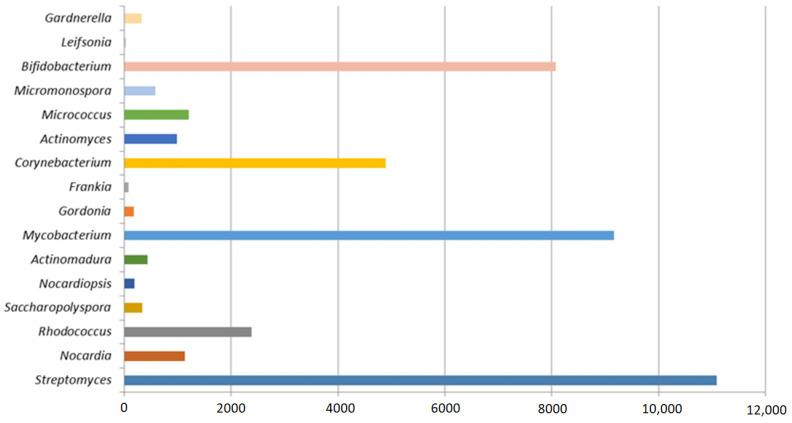
The total number of issued patents using actinomycetes (data on patent search in https://patentscope.wipo.int/ (front page), accessed on 25 March 2022).

The high biotechnological potential of this group of microorganisms was confirmed by patent analysis (Figure 1), with the largest number of valid patents using actinomycete genera such as *Streptomyces*, *Mycobacterium*, *Corynebacterium*, *Bifidobacterium*, and *Rhodococcus*.

Terpene biosynthesis by actinomycetes is an actual research area discussed in research and review publications. However, the specialized reviews are focused on certain genera of actinomycetes and/or groups of terpene derivatives [41,42], bacterial terpenome [43], and evolution and ecology of microbial terpenoids [44]. The present review aims at assessing the biosynthetic potential (*via* the patent analysis in particular) of various representatives of *Actinomycetes* as producers of a wide range of biologically active terpenoids, including hybrid metabolites (meroterpenoids). The data can be used to create technologies for the biocatalytic production of practically valuable terpene derivatives using actinomycetes.

In writing this review, various databases were used: scientific articles and reviews were searched through platforms such as Web of Science, Scopus, and NCBI, and WIPO (World Intellectual Property Organization, https://patentscope.wipo.int/, accessed on 25 March 2022) was used to search for patents. To fully cover the topic, the review includes patents and articles (from 2000 to 2022) dedicated to terpene biosynthesis by representatives of *Actinomycetes* (according to the modern classification). 

## 2. Terpene Biosynthesis by Actinomycetes

**Figure 2:**
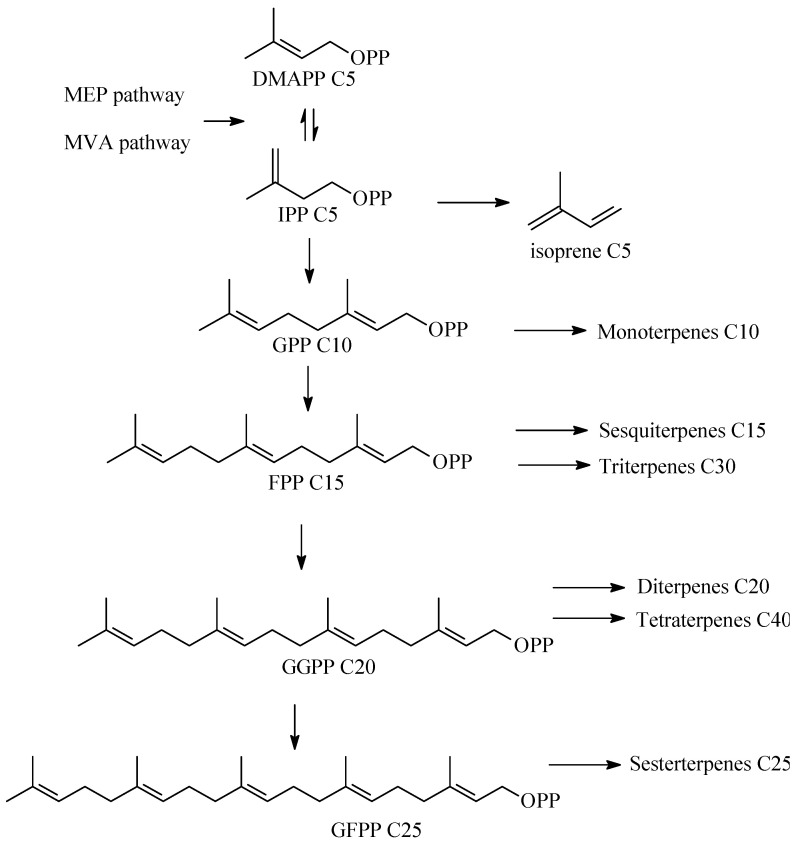
Biosynthesis of different terpenes. OPP: pyrophosphate group.

Terpene biosynthesis is one of the secondary metabolic pathways in actinomycetes, regulated by biosynthetic gene clusters (BGCs). BGCs include promoters, genes encoding carbon skeleton formation enzymes and post-modification enzymes, and regulatory genes. All terpenes are synthesized from the C5 isoprenoid precursors, namely isopentenyl diphosphate (IPP) and dimethylallyl diphosphate (DMAPP), which are converted to isoprenyl diphosphates of varying lengths by isoprenyl transferases (Figure 2). Further formation of terpenes is catalyzed by a group of enzymes, namely terpene synthases (cyclases) (TSs) catalyzing the cyclization of geranyl (GPP), farnesyl (FPP), geranylgeranyl (GGPP), and geranylfarnesyl (GFPP) diphosphates to yield mono-, sesqui-, di-, sester-, and triterpenes. Unlike the basic biosynthetic enzymes, bacterial TSs have low homology of conserved sequences, providing an extremely diverse group. The main feature of TSs is that one enzyme can produce dozens of hydrocarbon skeletons significantly different from each other. A number of remarkable reviews have been devoted to bacterial and plant terpene synthases [5,6,45,46]. Modification of the terpene skeleton is achieved through the addition of various functional groups mediated by specialized enzymes, mainly those from the cytochrome (P450s) family.

A variety of methods (bioinformatics, genetic, analytical, biochemical, molecular) are employed to study terpene biosynthesis by actinomycetes. Direct screening of compounds from the microbial cultivation medium and their subsequent identification is a basic method of searching for new terpene derivatives; however, it is labor- and time-consuming. Currently, recently developed “genome mining” methods, namely a bioinformatics search for TS genes using the BLAST program and web-based tools such as ClustSCAN, NP.searcher, GNP/PRISM, and antiSMASH, are used to search for actinomycetes capable of producing terpene derivatives. Simultaneous discovery of new compounds and biosynthetic genes and enzymes is one of the most important advantages of the coordinated use of genome analysis and direct analysis of the metabolites. Using this approach, a few dozen terpenes (many of which are unique), several new cyclization mechanisms, and more than 120 putative genes of bacterial terpene synthases have been discovered [47].

Methods of genetic modification (e.g., gene knockout, presumably responsible for the terpene synthesis; editing of individual sections of BGCs, in particular, by introducing additional native or engineered promoters; influence on the regulatory gene expression) and heterologous expression (e.g., cloning of the interest gene in bacteria that are not capable of synthesizing the target product) are used to confirm the functional activity of the studied genes. *E. coli* or mutant strains *Streptomyces avermitilis* SUKA 2–22 with deletion of all endogenous BGCs [48], *Streptomyces lividans* [49], *Streptomyces coelicolor*, *Streptomyces albus*, etc. [50,51], can serve as host bacteria. The transformants are used either for the direct terpene synthesis or for the production of recombinant proteins subsequently incubated with acyclic allyl diphosphate substrates. Molecular and biochemical methods allow studying the crystal structure, kinetic and mechanistic parameters of isolated and purified TSs and mechanisms of terpene cyclization [47]. In addition, omics technologies have been actively developed to search for secondary metabolites, terpenoids in particular, to study the diversity, distribution, and evolution of BGCs [52].

### 2.1. Terpene Derivatives Produced by Streptomycetes and Their Enzymes

The analysis of published data indicates that most of the identified actinomycete terpene derivatives are synthesized by streptomycetes. The spectrum of produced compounds varies from mono- to tetraterpenes and their derivatives.

#### 2.1.1. Mono- and Sesquiterpenes and Their Derivatives

The formation of monoterpenoids as secondary metabolites was registered for individual streptomycetes. *Streptomyces clavuligerus* ATCC 27064 have been shown to catalyze the formation of monoterpenoids cineole (**1**, eucalyptol) and linalool (**2**) [53,54,55]. Heterologous expression of terpene synthases bLinS и bCinS from *S. clavuligerus* ATCC 27064 in *E. coli* increased linalool (**2**) and 1,8-cineole (**1**) yields to 363 ± 57.9 and 116.8 ± 36.4 mg/L_org_, respectively, which exceeded the values obtained using plant enzymes. Furthermore, bLinS catalyzed the nerolidol (**3**) formation (159.1 ± 71.3 mg/L_org_) and acted as a mono- and sesquiterpene synthase (WO2018142109). The use of recombinant bLinS increased the nerolidol (**3**) and linalool (**2**) yields to 379 and 1054 ± 245.2 mg/L_org_, respectively [56] (WO2020234307; US20210238640). Two new nerolidol-type sesquiterpenoids rel-6*R*,7*R*,10*R*-6,10-epoxy-3,7,11-trimethyldodec-2-ene-1,7,11-triol (**4**), and rel-6*R*,7*R*,10*R*-7,10-epoxy-3,7,11-tri-methyldodec-2-ene-1,6,11-triol (**5**) were isolated from *S. scopuliridis* YIM 32460 [57].

2-Methylisoborneol (**6**) is an odorous irregular monoterpenoid identified in cultivation medium of some species of streptomycetes [58,59,60,61,62]. Using *S. coelicolor* A3(2) as an example, the two-gene cluster *sco7700*/*sco7701*, whose analogues were identified in *S. griseus*, *S. ambofaciens*, and *S. scabies*, was found to be responsible for 2-methylisoborneol (**6**) synthesis. Incubation of GPP with recombinant SCO7700A resulted in the production of a complex mixture of cyclic monoterpenes α-pinene (**7**), β-pinene (23%) (**8**), limonene (32%) (**9**), γ-terpinene (29%) (**10**), δ-terpinene (10%) (**11**), and trace amounts of monoterpene alcohols [63]. Köksal et al. (2012) determined the crystal structure of 2-methylisoborneol synthase from *S. coelicolor* A3(2) [64]. This enzyme was found to catalyze the formation of (1*R*)-(+)-camphor (**12**) from 2-fluorolinalyl diphosphate [65]. A non-oxidized bicyclic monoterpene 2-methyl-2-bornene (**13**) was identified among secondary metabolites of *S. exfoliatus* SMF19 [66].

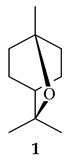

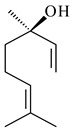

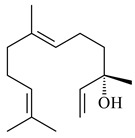

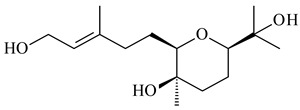
**1****2****3****4**
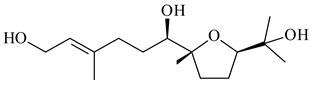

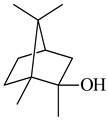

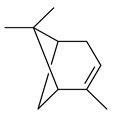

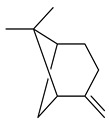
**5****6****7****8**
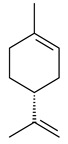

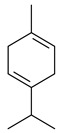

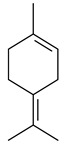

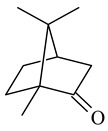

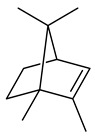
**9****10****11****12****13**

Two homologous genes *sc1* и *sc2* from *S. citricolor* NBRC 13005 were involved in the formation of monocyclic sesquiterpenoids (-)-germacradien-4-ol (**14**) and (-)-*epi*-α-bisabolol (**18**) with more than 85% yields [67]. A distinctive feature of germacradien-4-ol synthase is its high specificity and one terpenoid formed as the main product [68]. An uncharacterized TS of *S. pratensis* ATCC 33331 was identified as (+)-(1(10)*E*,4*E*,6*S*,7*R*)-germacradien-6-ol synthase and produced compound **19** [69], while terpene synthase Gd11olS from *S. coelicolor* A3(2) catalyzes FPP cyclization into germacradien-11-ol (**15**). Computer simulation combined with site-directed mutagenesis of Gd11olS changed the reaction direction with the formation of non-hydroxylated terpene isolepidozene (**20**) (88%) [70]. Along with the known germacradien-11-ol (**15**), new monocyclic sesquiterpenoids 1(10)*E*,5*E*-germacradiene-3,11-diol (**16**), 1(10)*E*,5*E*-germacradiene-2,11-diol (**17**), and roseosporol A (**21**) were identified from *S. griseus* wild type strain [71] and *S. roseosporus Lsr2*-deletion mutant strain [72], respectively. 1(10)*E*,5*E*-Germacradiene-3,11-diol (**16**) was detected among the secondary metabolites of *S. albolongus* YIM 101047 isolated from *Elephas maximus* feces [73].

Many streptomycetes are characterized by the formation of geosmin (**22**), a sesquiterpenoid causing a specific smell of moist soil [59,74,75,76]. Microbial methods of geosmin production by *S. albus* LBG-FXJ (CGMCC 4206), *S. fradiae* FJ-HX (CGMCC 4205), *Streptomyces* sp. QC-1 (CGMCC 4535), and *Streptomyces* sp. QC-2 (CGMCC 4536) have been patented (CN102719376; CN102719375; CN102181392; CN102719377). Genes and enzymes involved in geosmin biosynthesis were studied in the works of Cane et al. (2003–2008). Expression of recombinant protein SC9B1.20 (=SCO6073) from *S. coelicolor* A3(2) in *E. coli* resulted in Mg^2+^-dependent transformation of FPP to (4*S*,7*R*)-germacra-1(10)*E*,5*E*-dien-11-ol (**23**), a precursor of **22**, which indicates that the enzyme belongs to germacradienol/geosmin synthase [75]. Subsequently, germacradienol/germacrene D synthase was shown to be a bifunctional enzyme that, along with **22** (13%) and **23** (74%), catalyzed the formation of (−)-(7*S*)-germacrene D (**24**) (10%) and a hydrocarbon (3%) [77,78,79,80], which was later identified as (8*S*,9*S*,10*S*)-8,10-dimethyl-1-octalin (**25**) [79]. Genes *Sav2163* (*geoA*) and *spterp13*, analogs of *sco6073*, were found in *S. avermitilis* [74] and *S. peucetius* ATCC 27952 [81], respectively. Incubation of selina-4(15),7(11)-diene synthase from *S. pristinaespiralis* ATCC 25486 [82] and SAV_76 from *S. avermitilis* [83] with FPP produced trace amounts of germacrene B (**26**) and germacrene A (**27**). Recombinant SpS from *S. xinghaiensis* S187 catalyzed cyclization of FPP to germacrene D (**24**), germacrene A (**27**), and bicyclogermacrene (**28**) [84]. Germacrene D (**24**) was also isolated from the culture medium of *S. hygroscopicus* NRRL 15879 [66].

A new bicyclic sesquiterpenoid (5*S*,8*S*,9*R*,10*S*)-selina-4(14),7(11)-diene-8,9-diol (**29**) was produced by *Streptomyces* sp. QD518 [85]. Crystallographic, functional characteristics, and molecular mechanisms of selina-4(15),7(11)-diene synthase (SdS) from *S. pristinaespiralis* ATCC 25486 catalyzing the formation of **30** were described [82,86]. *Epi*-cubenol (**31**), a bicyclic cadinane sesquiterpenoid, was detected among terpenoids produced by *Streptomyces* sp. GWS-BW-H5 [53] and *S. albolongus* YIM 101047 [73]. Overexpression of *sgr6065* (*gecA*) from *S. griseus* IFO13350 in *S. lividans* TK21 led to (+)-*epi*-cubenol (**31**), while the *gecA*-knockout mutants lost this ability [87]. In the deuterated growth medium of *S. griseus* NBRC102592, the unique [2H_26_]-1-*epi*-cubenol, firstly obtained by fermentation, was synthesized [88]. *Streptomyces* sp. JMRC:ST027706 and *Streptomyces* sp. HKI0595 were isolated from mangrove trees *Bruguiera gymnorrhiza* and *Kandelia candel* and produced novel 11-hydroxy- (**32**), 12-hydroxy- (**33**) derivatives of **31** and 5,11-epoxy-10-cadinanol (**35**) [89] and five novel eudesmene-type sesquiterpenoids kandenols A-E (**36**–**40**) [90]. Kandenols A (**36**) and B (**37**) have a similar structure with plant eudesmenes, while kandenols C (**38**) and D (**39**) are unique due to the presence of hydroperoxide fragments. Kandenol E (**40**) is the first agarofurane isolated from bacteria. The strains *S. sanglieri* YIM 121209-2 [91], *S. anulatus* YIM 101882 [92], and *Streptomyces* sp. RM-14-6 [93] produced new 15-hydroxy-(+)-*epi*-cubenol (**34**), 5,11-epoxy-10-cadinanol (**35**), and isopterocarpolone (**41**), respectively. 

Two new eudesmane-type sesquiterpenoids 1α,6β,11-eudesmanetriol (**42**) and 11-eudesmene-1α,6β-diol (**43**) were isolated from *Streptomyces* sp. YIM 56130 [94]. Along with **42** and 4β,5β,7β,10α-5,11-eudesmanediol (**44**), *S. anulatus* YIM 101882 produced new sesquiterpenoids **45**–**47** and norsesquiterpenoids **48**–**50** [92]. New norsesquiterpenoids **51**–**57** were synthesized by *Streptomyces* sp. 0616208 [95], *Streptomyces* sp. XM17 [96], and *S. albolongus* YIM 101047 [73].

As a result of heterologous expression of *sscg_02150* and *sscg_03688* from *S. clavuligerus* ATCC 27074 in *E. coli*, TSs catalyzing the (−)-δ-cadinene (**58**) and (+)-T-muurolol (**59**) formation were isolated [97]. Along with (+)-T-muurolol (**59**) and 3α-hydroxy-T-muurolol (**60**), two new derivatives of **59**, namely 15-hydroxy- (**61**) and 11,15-dihydroxy (**62**) derivatives, were obtained from *Streptomyces* sp. M491 [98].

Purified dauc-8-en-11-ol synthase from *S. venezuelae* ATCC 10712 was shown to accept non-natural analogues of FPP, such as 10-methyl-FPP, 13-desmethyl-FPP, with the formation of methylated daucenol (**64**), widdrenol (**65**); nor-widdrenol (**66**); tenuifola-2,10-diene (**67**); and tenuifola-2,11-diene (**68**). The site-directed mutagenesis of the dauc-8-en-11-ol synthase resulted in a four-fold increase in the biosynthesis efficiency of the target terpenoid **63** [99]. Terpene synthases from *S. pristinaespiralis* ATCC 25486 [100], *S. clavuligerus* ATCC 27064, and *S. scabiei* 8722 [101] catalyzed the formation of (+)-(2*S*,3*S*,9*R*)-pristinol (**69**), new (+)-intermedeol (**70**), and (-)-neomeranol B (**71**), respectively.

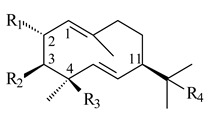

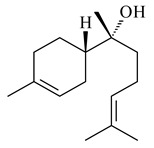

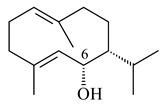

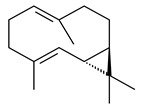
**14** R_1_=R_2_=H, R_3_=OH, R_4_=H **15** R_1_=R_2_=R_3_=H, R_4_=OH **16** R_1_=R_3_=H, R_2_=R_4_=OH **17** R_1_=OH, R_2_=R_3_=H, R_4_=OH**18****19****20**
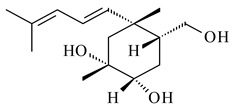

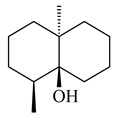

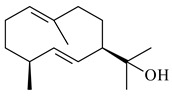

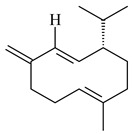
**21****22****23****24**
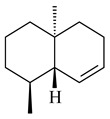

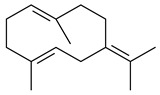

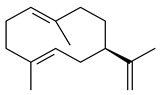

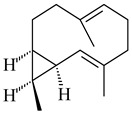
**25****26****27****28**
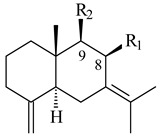

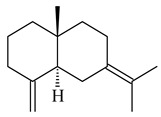

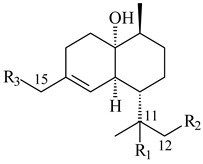

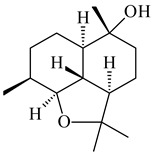
**29****30****31** R_1_=R_2_=R_3_=H **32** R_1_=OH, R_2_=R_3_=H **33** R_1_=R_3_=H, R_2_=OH **34** R_1_=R_2_=H, R_3_=OH**35**
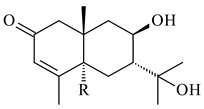

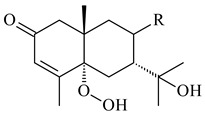

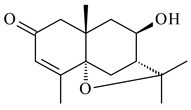

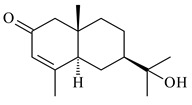
**36** R=H **37** R=OH**38** R=H **39** R=OH**40****41**



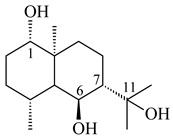



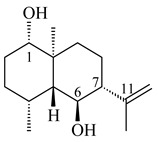



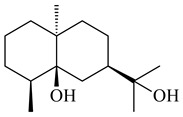


**42**

**43**

**44**


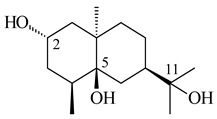



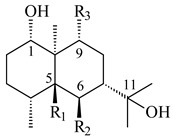



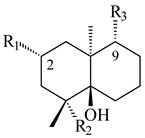



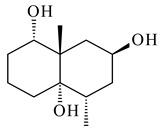


**45**
**46** R_1_=OH, R_2_=R_3_=H **47** R_1_=H, R_2_=R_3_=OH**48** R_1_=R_2_=H, R_3_=OH **49** R_1_=OH, R_2_=R_3_=H **50** R_2_=OH, R_1_=R_3_=H
**51**


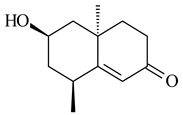



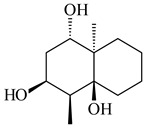



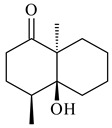



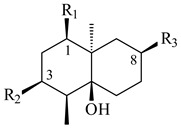


**52**

**5**
**3**

**54**
**55** R_1_=OH, R_2_=R_3_=H **56** R_1_=OH, R_2_=H, R_3_=OH **57** R_1_=H, R_2_=OH, R_3_=H 



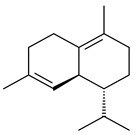



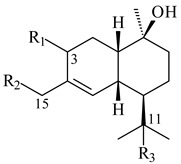



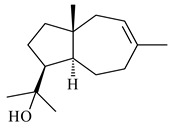



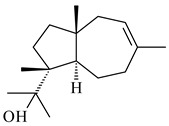


**58**
**59** R_1_=R_2_=R_3_=H **60** R_1_=OH, R_2_=R_3_=H **61** R_1_=R_2_=H, R_3_=OH **62** R_2_=R_3_=OH, R_1_=H
**63**

**64**


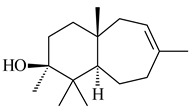



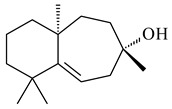



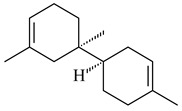



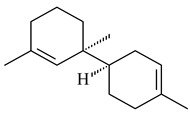


**65**

**66**

**67**

**68**


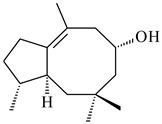



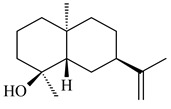



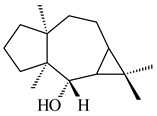



**69**

**70**

**71**



Cheng et al. (2020) studied the ability of streptomycetes to synthesize different volatile terpene derivatives, among which mono-, bi-, and tricyclic sesquiterpenoids were found [66]. *S. hygroscopicus* NRRL 15879 produced bicyclic sesquiterpenoids β-eudesmol (**72**), β-vatirenene (**73**), calamene (**74**), compound **75**, and tricyclic sesquiterpene β-cedrene (**76**). Additionally, the above strain catalyzed the formation of β-patchoulene (**77**), dehydro-β-agarofuran (**78**), and aromadendrene oxide-(2) (**79**). The monocyclic α-elemol (**80**), bicyclic sesquiterpene derivatives α-himachalene (**81**), β-eudesmol (**72**), α-muurolene (**82**), and a new 7β-hydroxy-7-*epi*-α-eudesmol (**84**) were derived from *S. parvulus* B1682, *S. clavuligerus*, *S. exfoliatus* SMF19, *S. aureofaciens* ATCC 12551 [66], and *S. sanglieri* YIM 121209-2 [91], respectively. Three new sesquiterpene synthases from *S. chartreusis* NRRL 3882 catalyzed the formation of germacradiene-11-ol (**15**), α-eudesmol (**83**), and α-amorphene (**85**) as major products and 10-*epi*-γ-eudesmol (**86**) as a minor product [102]. Incubation of recombinant TSs from *S. viridochromogenes* DSM 40736 with FPP yielded the products identified as 7-*epi*-α-eudesmol (**83**) and α-amorphene (**85**) [103].

Tricyclic humulane sesquiterpenoid (+)-isoafricanol (**87**) was identified among the volatile metabolites produced by *S. violaceusniger* Tü 4113. A recombinant (+)-isoafricanol synthase from *S. malaysiensis* DSM 4137 catalyzed the formation of **87** (95%) and trace amounts of african-1-ene (**88**) and african-2(6)-ene (**89**) [104]. Incubation of recombinant SAV_76 of *S. avermitilis* with FPP in the presence of Mg^2+^ resulted in avermitilol (**90**), a novel sesquiterpene alcohol, and viridiflorol (**91**). Transformants of *S. avermitilis* SUKA17 containing copies of the *sav76* gene and the native *rpsJp* (*sav4925*) promoter afforded the new ketone avermitilone (**92**) along with previously obtained compounds [83].

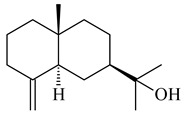

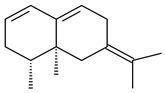

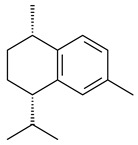

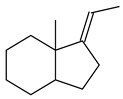
**72****73****74****75**
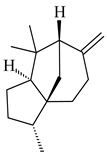

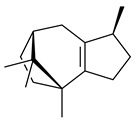

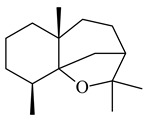

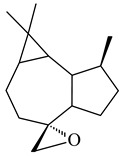
**76****77****78****79**
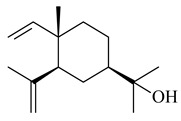

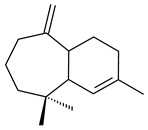

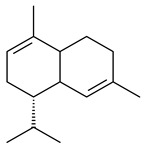

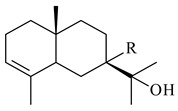
**80****81****82****83** R=H **84** R=OH
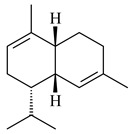

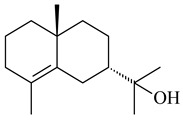

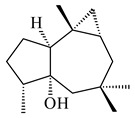

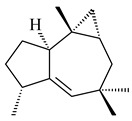
**85****86****87****88**
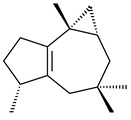

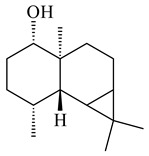

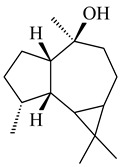

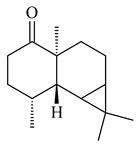
**89****90****91****92**

Tricyclic sesquiterpene β-caryophyllene (**93**) was identified among the volatile organic compounds produced by *S. yanglinensis* 3-10 [62]. The formation of (+)-caryolan-1-ol (**94**), an oxidized derivative of β-caryophyllene (**93**), was observed during the cultivation of wild-type or genetically modified strains of streptomycetes [73,105,106,107] (WO2018062668). Along with known 9α-hydroxy- (**95**), 9β-hydroxy- (**96**), novel 7α-hydroxy- (**97**), 10-hydroxy- (micaryolane A) (**98**), and 15-hydroxy- (micaryolane B) (**99**) derivatives of **94** were isolated from *Streptomyces* sp. YIM 56130 [94], *Streptomyces* sp. AH25 [108], *S. anulatus* YIM 101882 [92], and *S. albolongus* YIM 101047 [73]. Bacaryolanes A-C (**100**–**102**), enantioisomers of plant caryolans, were separated from the fermentation broth of *Streptomyces* sp. JMRC:ST027706 [109] and *S. anulatus* YIM 101882 [92].

*Epi*-isozizaene (**103**), tricyclic sesquiterpene, was generated by several *Streptomyces* species and initially sparked interest as a candidate jet fuel on account of having a specific energy similar to that of jet fuel A-1 [110,111]. Heterologous *epi*-isozizaene synthase from *S. coelicolor* A3(2) and pentalenene synthase from *Streptomyces* sp. UC5319 produced **103**, pentalenene (**107**) and α-isocomene (**108**) [111]. Using the genetic engineering techniques increased the yields of **108**, **103,** and **107** in *E. coli* to 77.5 mg/L, 727.9 mg/L, and 780.3 mg/L, respectively, while the yield of **107** was improved to 344 mg/L in *Saccharomyces cerevisiae* (US20200239796).

*Epi*-isozizaene synthase (*sco5222*) of *S. coelicolor* A3(2) catalyzed multi-step cyclization of FPP to **103**, which is oxidized by P450 (*sco5223*) to albaflavenone (**109**), a broad-spectrum antibiotic [112,113,114], detected in the culture medium of some species of streptomycetes [115,116,117]. Genome-wide analysis of *S. spectabilis* NRRL-2792 found the albaflavenone biosynthetic gene cluster [118]. *S. avermitilis* SUKA16 transformant, which expresses *sav3032* (ortholog *sco5222*) and promoter *rpsJp* (*sav4925*) from the native strain *S. avermitilis*, accumulated **103**, (4*R*)-albaflavenol (**104**), (4*S*)-albaflavenol (**105**), albaflavenone (**109**), and a new compound 4β,5β-epoxy-2-*epi*-zizaan-6β-ol (**110**) [119]. New sesquiterpenoids identified as albaflavenol B (**106**) and albaflavenoid (**111**) were isolated from *Streptomyces* sp. Lv-4-26 [120] and *S. violascens* YIM 100225 [121], respectively.

Twenty-six site-directed mutants of the *S. coelicolor* A3(2) *epi*-isozizaene synthase catalyzed the formation of acyclic (**119**–**121**), mono- (**122**–**125**), bi- (**126**–**130**), and tricyclic (**110**, **83**, **131**–**135**) sesquiterpenes, which makes this enzyme a universal platform for obtaining various terpene derivatives [110,122] (WO2015120431). New tricyclic sesquiterpenoids strepsesquitriol (**136**) and bungoene (**137**) were obtained from *Streptomyces* sp. SCSIO 10355 [123] and *S. bungoensis* DSM 41781 [124], respectively.

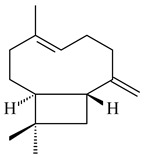

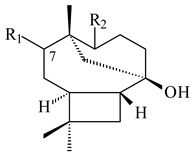

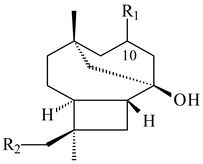

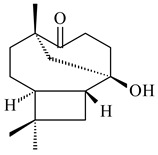
**93****94** R=H **95** R_1_=H, R_2_=αOH **96** R_1_=H, R_2_=βOH **97** R_1_=αOH, R_2_=H**98** R_1_=OH, R_2_=H **99** R_2_=OH, R_1_=H**100**
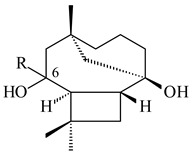

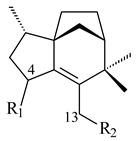

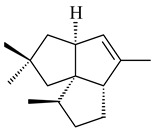

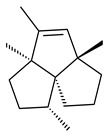
**101** R=αH **102** R=βH**103** R_1_=R_2_=H **104** R_1_=αOH, R_2_=H **105** R_1_=βOH, R_2_=H **106** R_1_=αOH, R_2_=OH**107****108**
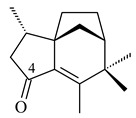

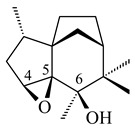

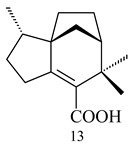

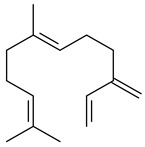
**109****110****111****112**
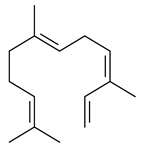

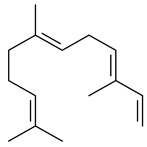

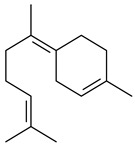

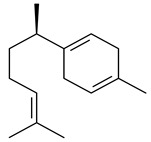
**113****114****115****116**
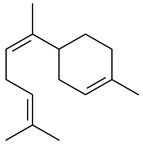

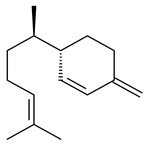

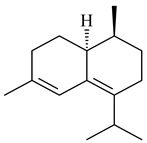

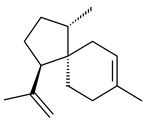
**117****118****119****120**
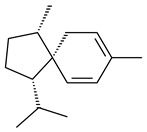

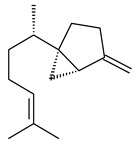

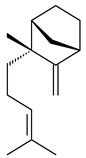

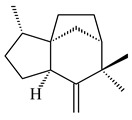
**121****122****123****124**
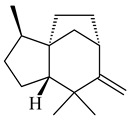

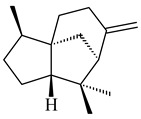

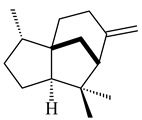

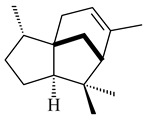
**125****126****127****128**
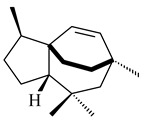

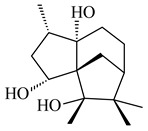

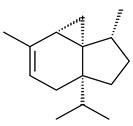
**129****130****131**

Pentalenolactone (**132**) is a tricyclic sesquiterpenoid antibiotic, derived from pentalenene (**107**) and synthesized by more than 30 *Streptomyces* species. The resistance of streptomycetes to **132** was found to be determined by the *gap1* gene (*sav2990*). Pentalenene synthase was first isolated from *S. exfoliatus* UC5319 in the 1990s. Exemplified by *S. avermitilis*, *S. exfoliatus* UC5319, and *S. arenae* TÜ469, the metabolic pathways of pentalenolactone synthesis were studied. The 13.4 kb BGC comprising 13 unidirectionally transcribed open reading frames (ORFs) (*sav2990*–*sav3002*) was shown to be responsible for the pentalenolactone (**132**) synthesis. The cyclization of FPP to **107** is catalyzed by PtlA (*sav2998*) [125]. Its further oxidation involves PtlI (*sav2999*) with the formation of 1-deoxypentalen-13-ol (**133**), 1-deoxypentalen-13-al (**134**), and 1-deoxypentalenic acid (**136**) [126], while its oxidation with PtlH hydroxylase (*sav2991*), PtlF dehydrogenase (*sav2993*) and PenD, PntD, or PtlD resulted in the formation of (-)-11β-hydroxy-1-deoxypentalic acid (**137**) [127], 1-deoxy-11-oxopentalenic acid (**138**) [128], and pentalenolactones D (**140**), E (**141**) and F (**142**) [129], respectively. The *penM* and *pntM* genes were found to be responsible to final step in pentalenolactone biosynthesis [130]. Pentalenolactone biosynthesis in *S. exfoliatus* UC5319 and *S. arenae* TÜ469 is regulated by the orthologous proteins PenR and PntR [131]. Jiang et al. (2009) described a new direction of the pentalenolactone biosynthetic pathway involving the oxidation of **138** by PtlE (*sav2994*) to neopentalenolactone D (**143**), and its subsequent conversion to neopentalenolactone E (**144**), compound PL308 (**145**), hydroxyl derivatives (**139**) and (**146**), an oxidized lactone (**147**), and seco-acids **148** and **149** [132].

Pentalenic acid (**135**), a co-metabolite of **132** and **143**, is formed due to the oxidation of **136** by cytochrome CYP105D7 (*sav7469*) [133]. Genome-wide analysis of *Streptomyces* sp. NRRL S-4 identified a biosynthetic cluster of pentalenolactone type terpenes: 1-deoxy-8α-hydroxypentalic acid (**150**) and 1-deoxy-9β-hydroxy-11-oxopentalenic acid (**151**) [134].

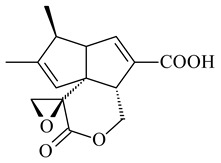

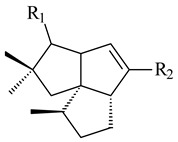

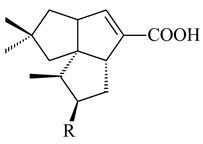

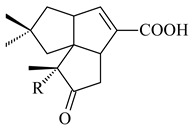
**132****133** R_1_=H, R_2_=CH_2_OH **134** R_1_=H, R_2_=CHO **135** R_1_=OH, R_2_=COOH**136** R=H **137** R=OH**138** R=H **139** R=OH
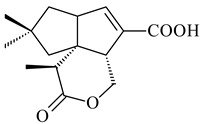

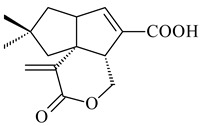

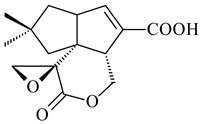

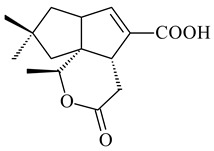
**140****141****142****143**
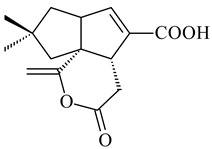

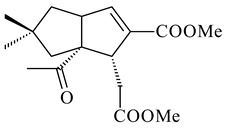

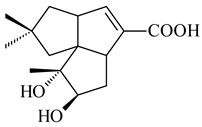

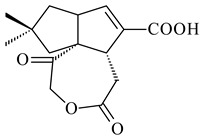
**144****145****146****147**
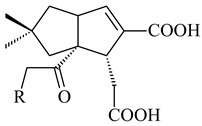

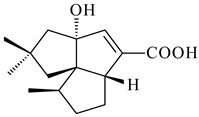

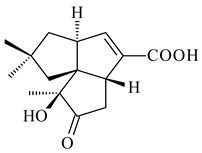

**148** R=H **149** R=OH**150****151**


The *S. avermitilis* SUKA22 transformant with *sclav_p1407* afforded eight sesquiterpenes, with the tricyclic isohirsut-1-ene (cucumene, **152**) being the main product. With that, *slt18_1880* of *S. lactacystinaeus* OM-6159 was responsible for the formation of isohirsut-4-ene (**153**). Isohirsut-1-ene (**152**) and isohirsut-4-ene (**153**) are linear triquinane sesquiterpenes that have never been isolated from bacteria or any other source before [135] (WO2015022798). Using computer modeling, cucumene synthase B5GLM7, the first TS involved in the synthesis of linear triquinane, was identified in *S. clavuligerus* ATCC 27604 [136], and its crystal structure was later described [137]. The recombinant sesquiterpene synthase from *S. lincolnensis* NRRL 2936A produced a novel tetracyclic sesquiterpene isoishwarane (**154**) with a unique structure [138].

The recombinant SpS from *S. xinghaiensis* S187 converted 10,11-dehydro-FPP into sesquiterpenes isopentylkelsoene (**157**) and spat-13-ene (**161**). Moreover, it transformed GGPP into new diterpenes prenylkelsoene (**155**), spata-13,17-diene (**158**), cneorubin Y (**159**), and GFPP into new sesterterpenes geranylkelsoene (**163**) and prenylspata-13,17-diene (**160**). This reaction features of SpS proved that this TS exhibited sesqui-, di-, and sesterterpene synthase activity [84].

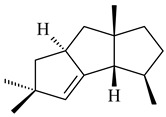

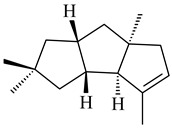

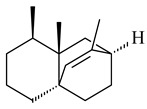

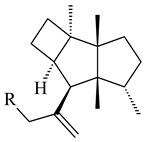
**152****153****154****155** R=prenyl **156** R=geranyl **157** R=*i*pent
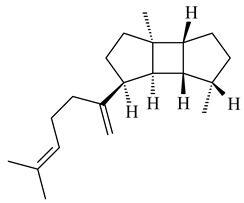

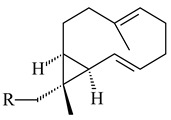

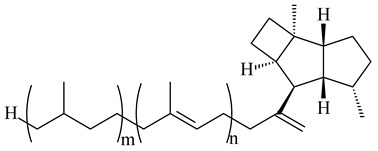
**158****159** R=prenyl**160** m=0, n=2 **161** m=1, n=0

#### 2.1.2. Diterpenes and Their Derivatives

Two unique terpene cyclases DtcycA and DtcycB from *Streptomyces* sp. SANK 60404 were described as responsible for the formation of cembrene C (**162**), (*R*)-nephthenol (**163**), (*R*)-cembrene A (**164**), and a new compound identified as (4*E*,8*E*,12*E*)-2,2,5,9,13-pentamethylcyclopentadeca-4,8,12-trien-1-ol (**165**) [139]. 

Co-cultivation of *S. cinnabarinus* PK209 with *Alteromonas* sp. KNS-16 induced the formation of a diterpenoid lobocompactol (**166**) [140]. The ability of streptomycetes to synthesize new eunicellane-type diterpenoids was proved. *Streptomyces* sp. CL12-4 [141] and *S. albogriseolus* SY67903 [142] produced unique benditerpenoic acid (**167**) and microeunicellols A (**168**), B (**169**), respectively. Enzymatic and mechanistic characteristics of the benditerpenoic acid synthase from *Streptomyces* sp. CL12-4 were described in the article [143].

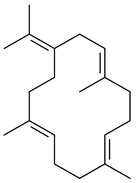

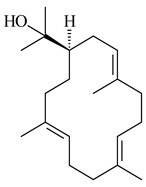

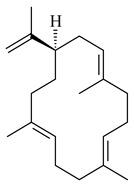

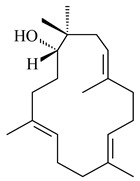
**162****163****164****165**
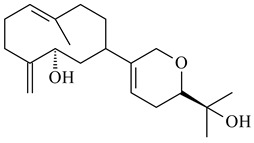

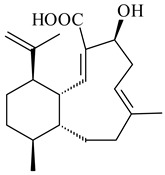

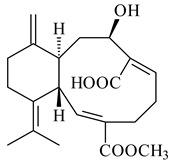

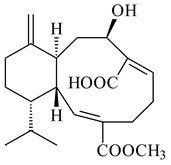
**166****167****168****169**

The transformants of *S. avermitilis* SUKA22 containing *CldD*/*CldB*, *CldB*/*SCLAV_p0490*, *SCLAV_p0490*/*CldD,* and *SCLAV_p0490*/*SCLAV_p0491* genes of diterpene synthases from *S. clavuligerus* ATCC 27064 produced labdane-type diterpenoids. The diterpene derivatives were identified as labda-8(17),12(*E*),14-triene ((*E*)-biformene, **170**), labda-8(17),13(16),14-triene (**172**), ladba-7,12(*E*),14-triene (**173**), and a new compound labda-7,13(16),14-triene (**174**) [144]. Centeno-Leija et al. (2019) described the X-ray crystal structure of (*E*)-biformene synthase isolated from *S. thermocarboxydus* K155 for the first time. The (*E*)-biformene synthase was encoded by the *LrdC*, which was identified as part of the *LRD* cluster [145,146]. Transformants of *S. coelicolor* M1152, *S. peucetius* var. *caesius* and *S. avermitilis* SUKA22 having the *LRD* cluster generated **170** [147]. *Streptomyces* sp. KIB 015 produced four new labdane-type diterpenoids, labdanmycins A–D (**175**–**178**), while the *labE* gene deletion led to the formation of raimonol (**171**), their biogenetic precursor [148]. The formation of compound **171** was also observed upon insertion of the *rmn* cluster from *S. anulatus* GM95 to *S. avermitilis* SUKA22. The transformants *S. avermitilis* SUKA22 [149] and *S. cyslabdanicus* K04-0144*Δcld* [147] containing the *cld* or *lrdABDC* clusters produced (7*S*,8*S*,12*E*)-8,17-epoxy-7-hydroxylabda-12,14-diene (**179**).

The diterpene synthase Stt4548 from *Streptomyces* sp. PKU-TA00600 catalyzed the *normal*-copalyl diphosphate (CPP) cyclization to isopimara-8(9),15-diene (**180**) [150]. Both strains *Streptomyces* sp. KO-3988 [151] and *Streptomyces* sp. SN194 [152] synthesized diterpenoid 3-hydroxypimara-9(11),15-diene (viguiepinol, **181**) via the formation of *ent*-CPP (**183**) and pimara-9(11),15-diene (**182**) as intermediates.

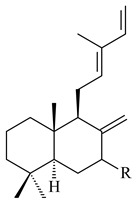

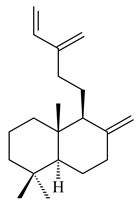

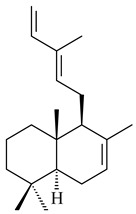

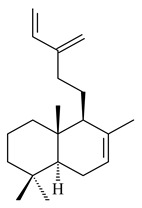
**170** R=H **171** R=OH**172****173****174**
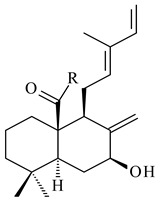

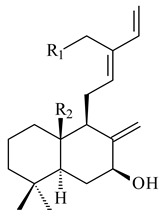

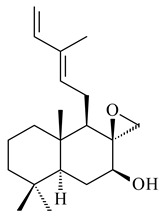

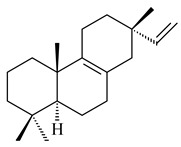
**175** R=H **176** R=OH**177** R_1_=H, R_2_=CH_2_OH **178** R_1_=OH, R_2_=CH_3_**179****180**
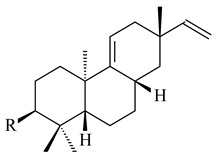

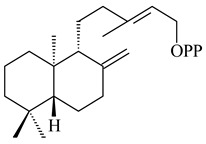

**181** R=OH **182** R=H**183**


The biosynthetic cluster responsible for synthesis of tricyclic diterpenoid cyclooctatin (**184**) was found in *S. melanosporofaciens* MI614-43F2. This cluster consists of four genes, *cotB1*-*cotB4*, encoding GGDP synthase, CotB2 terpene cyclase, and two P450 cytochromes. The incubation of recombinant CotB2 with GGDP resulted in the formation of cyclooctat-9-en-7-ol (**187**) [153]. Later, the crystal structure and mechanistic characteristics of CotB2 were described [154,155,156,157]. A mutant of diterpene synthase CotB2 (W288G) was found to produce (1*R*,3*E*,7*E*,11*S*,12*S*)-3,7,18-dolabellatriene (**188**), but not the native product **187** [158]. Recombinant *E. coli* carrying the *CotB3* or *CotB4* duet vector in combination with *AfR-Afx* gene cassettes from *S. afghaniensis* produced **184** with a 43-fold increase (up to 15 mg/L) compared with the native producer. Moreover, CotB3 was found to be able to hydroxylate (−)-casbene (**189**) to form sinularcasbane D (**190**) [159]. New 16,17-dihydroxy- (**185**) [160], 17-hydroxy- (**186**) [161,162] and 18-acetyl- (**191**), 5-dehydroxy- (**192**), and 5,18-dedihydroxy- (**193**) [163] derivatives of **184** were isolated from *Streptomyces* sp. LZ35, *Streptomyces* sp. MTE4a, *Streptomyces* sp. M56, and *Streptomyces* sp. ZZ820, respectively. Three new fusicoccane-type diterpenoids, 12α-hydroxy- (**194**), 12β-hydroxy- (**195**), and 14-hydroxycyclooctatin (**196**), were separated from the fermentation broth of *S. violascens* YIM 100212 isolated from the feces of *Ailuropoda melanoleuca* [164]. The formation of new tricyclic diterpene lydicene (**197**) was observed using the recombinant TS StlTC, with unique UbiA-type diterpene cyclases, from *S. lydicus* [165].

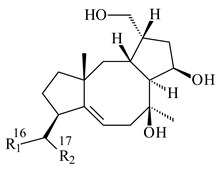

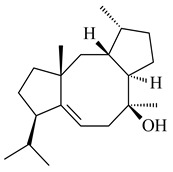

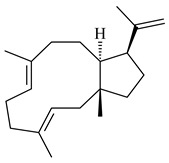

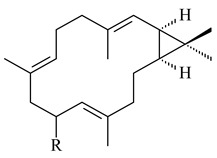
**184** R_1_=R_2_=CH_3_ **185** R_1_=R_2_=CH_2_OH **186** R_1_=CH_3_, R_2_=CH_2_OH**187****188****189** R=H **190** R=βOH
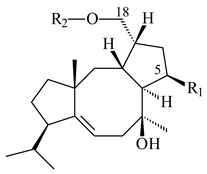

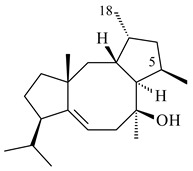

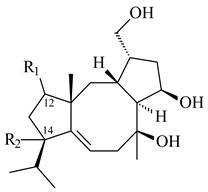

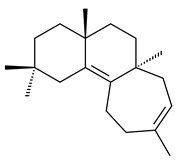
**191** R_1_=OH, R_2_=COCH_3_ **192** R_1_=R_2_=H**193****194** R_1_=αOH, R_2_=H **195** R_1_=βOH, R_2_=H **196** R_1_=H, R_2_=αOH**197**

Genome mining of *S. venezuelae* ATCC 15439 revealed *ven*, a silent biosynthetic cluster responsible for the synthesis of diterpenoids venezuelaenes A (**198**) and B (5-oxo-venezuelaene A) (**199**) with a unique 5-5-6-7 tetracyclic skeleton [166]. Rabe et al. (2017) performed a mechanistic study of two diterpene cyclases, spiroviolene synthase from *S. violens* NRRL ISP-5597 and tsukubadiene synthase from *S. tsukubaensis* NRRL 18488, which catalyze the formation of **200** and **201**. Although the structures of **200** and **201** are significantly different, the cyclization mechanisms of both enzymes proceed through the same initial cyclization reactions, which proved their phylogenetic similarity [167,168]. The generation of a new tetracyclic diterpene cattleyene (**202**) was observed using the recombinant TS CyS from *S. cattleya* NRRL 8057 [169].

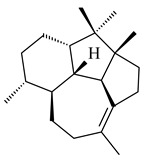

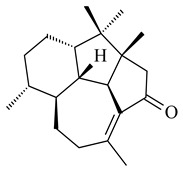

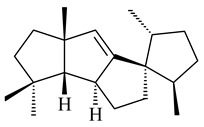
**198****199****200**
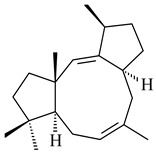

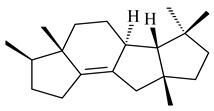

**201****202**


Based on the large-deletion mutant *S. avermitilis* SUKA22, the transformants catalyzing the formation of terpene derivatives with various structures were created. The expression of *sclav_p1169* and *sclav_p0765* from *S. clavuligerus* ATCC 26074 led to the formation of monocyclic prenyl-β-elemene (**203**), prenylgermacrene B (**204**), bicyclic clavulatriene A (**205**), clavulatriene B (**206**) or bicyclic isoelisabethatriene B (**207**), tetracyclic hydropyrene (**208**), and hydropyrenol (**209**). The transformant carrying *slt18_1078* from *S. lactacystinaeus* OM-6159 catalyzed a tricyclic diterpene cyclooctat-7(8),10(14)-diene (**210**). The *stsu_20912* gene from *S. tsukubaensis* NRRL 18488 was responsible for the synthesis of **201**, while the transformant with *nd90_0354* from *Streptomyces* sp. ND90 synthesized tricyclic odyverdienes A (**211**) and B (**212**). The derived diterpenoids are novel compounds with unique hydrocarbon skeletons [135] (WO2015022798). Under normal conditions, a hydropyrene synthase from *S. clavuligerus* ATCC 27064 produced hydropyrene (**208**, up to 52%) and hydropyrenol (**209**, up to 26%), and minor amounts of isoelisabethatrienes A (**213**) and B (**207**), biosynthetic precursors of pseudopterosins with pronounced anti-inflammatory activity. An increase in the yield of **213** and **207** to 41.91 ± 1.87 mg/L was achieved using a genetically modified hydropyrene synthase [170] (WO2022003167).

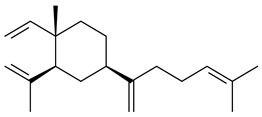

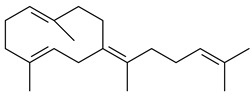

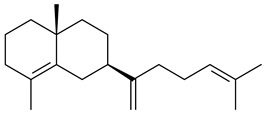
**203****204****205**
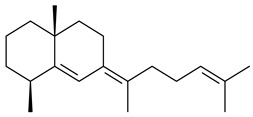

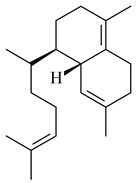

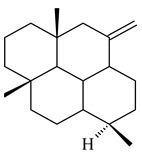

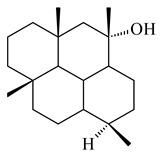
**206****207****208****209**
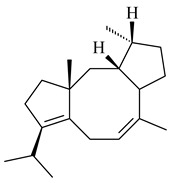

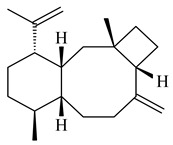

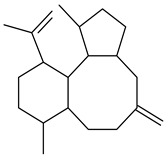

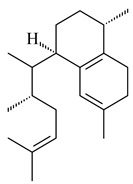
**210****211****212****213**

#### 2.1.3. Sester-, Tri-, and Tetraterpenes and Their Derivatives

Unlike sesqui- and diterpenes, the formation of terpene derivatives with a chain length of more than 20 carbon atoms was observed only for individual strains of *Streptomycetes*. Sesterterpene cyclases were isolated from *S. somaliensis* ATCC 33201™ and *S. mobaraensis* NBRC 13819 (=NRRL B-3729) and generated new somaliensenes A (**214**) and B (**215**) [171], sestermobaraenes A–F (**216**–**221**), and sestermobaraol (**222**) [172], respectively.

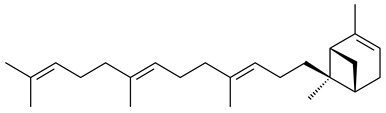

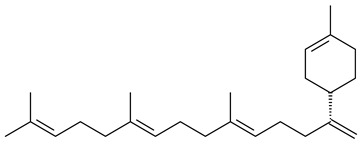
**214****215**
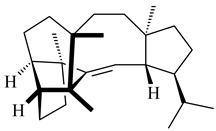

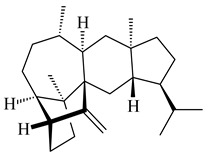

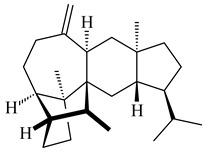
**216****217****218**
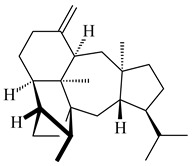

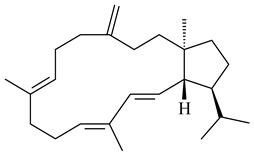

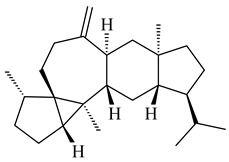
**219****220****221**
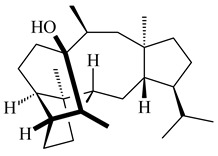


**222**



The heterologous expression of *hopA* and *hopB* (encoding squalene/phytoene synthases) and *hopD* (encoding farnesyl diphosphate synthase) from *S. peucetius* ATCC 27952 in *E. coli* provided an acyclic triterpene squalene (**230**) with a yield of 11.8 mg/L [173]. Another acyclic triterpene, botryococcene (**231**), was produced by activating the Fur22 regulator and simultaneous expression of the biosynthetic genes of *S. reveromyceticus* SN-593. The yield of the target product was 0.3 g/L, which is comparable to the levels of other microbial producers [174].

Hopanoids are unusual pentacyclic triterpenes present in bacterial species. Hop-22(29)-ene (**290**) was isolated from wild-type [175,176] and genetically modified strains of streptomycetes [72,177]. A genome-wide analysis of *S. scabies* 87–22 detected a hopanoid biosynthetic cluster responsible for the synthesis of **232** [178]. The squalene-hopene cyclase (*spterp25*) catalyzing the complex cyclization of **230** to the pentacyclic triterpene **232** was described for *S. peucetius* ATCC 27952 [179].

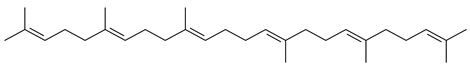

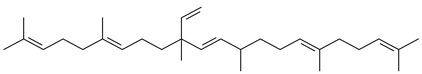
**223****224**
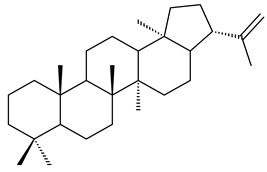

**225**


#### 2.1.4. Hybrid Metabolites (Meroterpenoids)

Meroterpenoids are products of mixed biosynthetic origin that consist of terpenoid scaffold combined with polyketide, alkaloid, phenol, or amino acid. According to their different biosynthetic origins, meroterpenoids can be divided into two groups, polyketide and non-polyketide terpenoids. Meroterpenoids have attracted researchers’ attention due to their unusual chemical structures and a wide range of biological properties [180]. 

Naphthoquinone-based meroterpenoids are large chemically diverse group including napyradiomycins, merochlorins, marinones, furaquinocins, etc., some of which have a high therapeutic potential. Naphthoquinone-based meroterpenoids derived from streptomycetes are described in the review published in 2020 [181], so our review highlights the most active producers and the derivatives with promising biological activity, as well as compounds isolated after 2020.

Biosynthesis of naphthoquinone-based meroterpenoids includes regioselective addition of aromatic polyketide (1,3,6,8-tetrahydroxynaphthalene) to a terpene diphosphate catalyzed by ABBA prenyltransferase (PTase). After the initial prenylation, oxidation, halogenation and cyclisation steps occur. Genome mining of streptomycetes as producers of naphthoquinone-based meroterpenoids led to the discovery of unique prenyltransferase and vanadium-dependent haloperoxidase (VHPO) enzymes, which differ significantly from those previously described for algae and fungi [182,183]. For instance, the high-resolution crystal structures of two homologous members of the VHPO family associated with napiradiomycin biosynthesis, NapH1 and NapH3, were characterized [184]. 

Furaquinocins A (**226**) and B (**227**) were first isolated from the culture broth of *Streptomyces* sp. KO-3988 [185] and *Streptomyces* sp. strain CLl 90 (WO2006081537). Later, analogues of these compounds (**228**–**231**, **234**, **235**) [186] and the *fur* cluster responsible for furaquinocin biosynthesis were determined [187]. Among secondary metabolites derived from *Streptomyces* sp. TBRC7642 new furaquinocin I (**232**), streptolactone (**239**) and previously identified furaquinocins B (**227**), D (**229**), and murayaquinone (**240**) were described [188]. Furaquinocins I (**232**), J (**233**), JBIR-136 (**236**), and furaquinocins K (**237**) and L (**238**) were obtained from genetically engineered *S. reveromyceticus* SN-593 [189], *Streptomyces* sp. 4963H2 [190], and *Streptomyces* sp. Je 1-369 [191].

*Streptomyces* sp. CNH-189 produced unique halogenated meroterpenoids, merochlorins A–J (**241**–**250**) and meroindenon (**251**) [192,193,194], of which biosynthesis determined the presence of *mcl* gene cluster with VHPO genes [182]. Flaviogeranin A (**252**) is promising neuroprotective agent produced by *Streptomyces* sp. RAC226 [195]. Along with **252**, six flaviogeranin congeners or intermediates (**253**–**258**), including novel flaviogeranins B1 (**255**), B (**253**), containing an amino group, and flaviogeranin D (**256**), were derived from *Streptomyces* sp. B9173 [196].

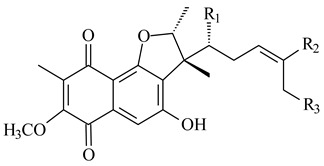

R_1_R_2_R_3_**226**OHCH_3_OH**227**OHCH_2_OHH**228**HCH_3_H**229**OHCH_3_H**230**HCH_2_OHH**231**OHCH_2_OHOH**232**OHCOOHH**233**OHCONH_2_H
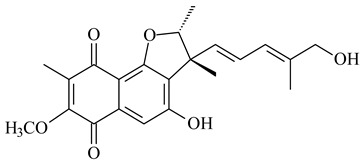

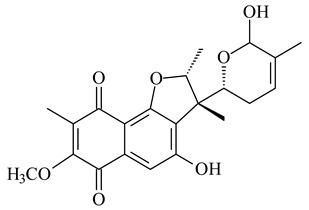
**234****235**
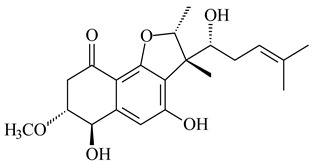

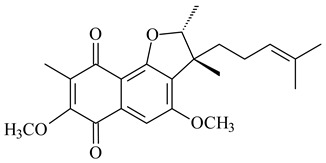
**236****237**
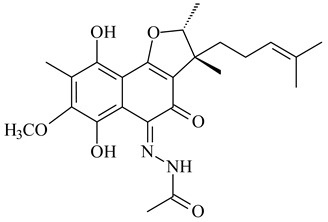

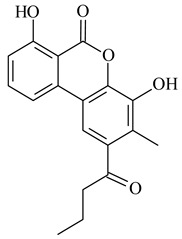

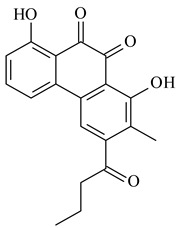
**238****239****240**
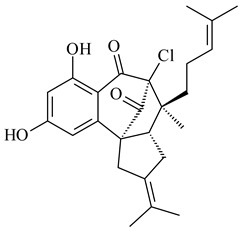

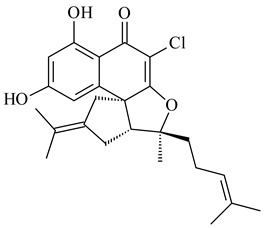

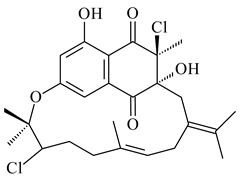
**241****242****243**
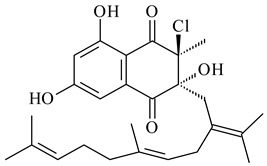

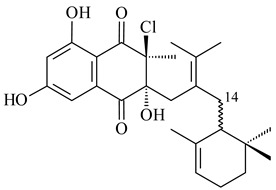
**244****245** **246**
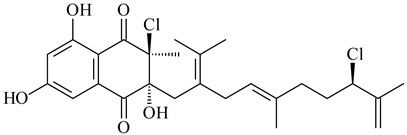

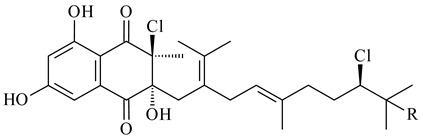
**247****248** R=OH **249** R=Cl
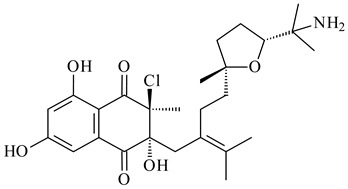

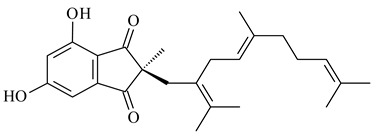
**250****251**
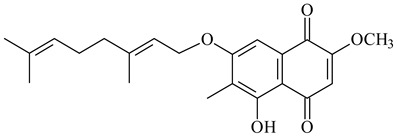

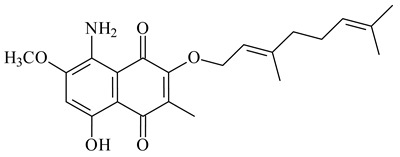
**252****253**
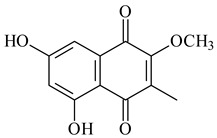

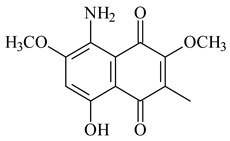

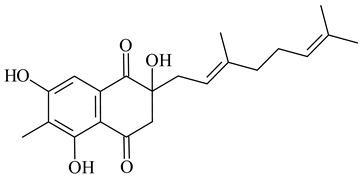
**254****255****256**
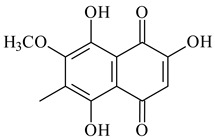

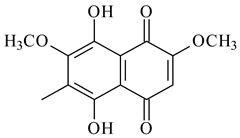

**257****258**


Naphthoquinone-based meroterpenoids naphterpin (**259**) and related compounds (**260**–**263**) were produced by *Streptomyces* sp. CNQ-509 and *Streptomyces* sp. CL190 (WO2006081537) and displayed pronounced antioxidant effect [197,198,199]. The napyradiomycins are a large group of unique meroterpenoids with different halogenation patterns and a monoterpenoid subunit attached to C10a. Napiradiomycins were first isolated from *Chainia rubra* in 1986 (later transferred to the genus *Streptomyces*), and more than 50 analogous compounds have been identified to date. They have been arranged into three main types according to their structural features: Type A with a linear terpene chain; Type B with the side chain cyclized to a cyclohexane ring; and Type C with monoterpenoid subunit cyclized between C7 and C10a of the naphthoquinone core to form a 14-membered ring.

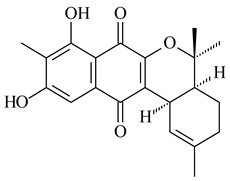

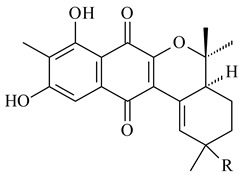

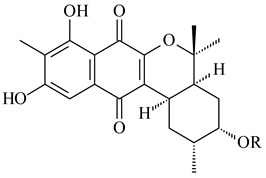
**259****260** R=βOH **261** R=αOH**262** R=H **263** R=COCH_3_

Among napyradiomycins produced by *Streptomyces* sp. YP127 [200], *Streptomyces* sp. CA-271078 [201], *S. antimycoticus* NT17 [202,203], and *Streptomyces* sp. SCSIO 10428 [204], biologically active napyradiomycin A1 (**264**) and its Br-containing (**266**) derivative were isolated. Chemical analysis of a crude extract of *Streptomyces* sp. YP127 detected a series of napyradiomycins, in particular 16*Z*-19-hydroxynapyradiomycin A1 (**265**) possessed the high anti-inflammatory and antioxidant activities [205]. Along with **264**, *Streptomyces* sp. CNQ-329, CNH-070 [206], and *Streptomyces* sp. SCSIO 10428 [204] produced napyradiomycins B type **273**, **274**, **275**, **284**, and the later strain also catalyzed the formation of bicyclic naphthomevalin (**289**). Napyradiomycins of A (**265**, **269**) and B (**275**) types as well as SF2415B3 (**269**), A80915A (**277**) carrying additional methyl group at C7 and their 4-dehydro-4a-dechloro- (**270**, **276, 282**) derivatives were isolated from *S. aculeolatus* PTM-029 and PTM-420 [207]. *Streptomyces* sp. CNQ-525 produced antibacterial or cytotoxic napyradiomycins **277**, **280**–**283** [208] and Br-containing **271** [209]. Napyradiomycins 7-demethyl SF2415A3 (**272**) and 7-demethyl A80915B (**285**) containing diazonium group as well as *R*-3-chloro-6-hydroxy-8-methoxy-α-lapachone (**286**) were derived from *S. antimycoticus* NT17 [202]. Napyradiomycin D1 (**287**) was derived from *Streptomyces* sp. CA-271078 [203] and displayed an unprecedented 14-membered cyclic ether ring between the prenyl side chain and the chromophore, thus representing the first member of a new type of napyradiomycins. The biosynthetic methods for obtaining of napyradiomycins A1 (**264**), B1 (**273**), A4 (**267**), A80915H (**290**), A80915G (**291**), naphthomevalin (**289**) by *S. kebangsaanensis* WS-68302 (CN114805278); A80915A (**277**), A80915B (**278**), A80915D (**279**), A80915G (**291**) by *S. aculeolatus* A80915 (NRRL 18422) (EP0376609); and 3-dechloro-3-bromonapyradiomycin A1 (**266**) by *Streptomyces* sp. SCSIO 10428 (CN105399721) were patented.

Four new sesquiterpene naphthoquinones, marfuraquinocins A–D (**292**–**295**), were isolated from the fermentation broth of *S. niveus* SCSIO 3406 [210].

Teleocidin B (**296**) is a well-known naturally occurring tumor promoter. Since the isolation of **296** in the early 1960s [211], more than 44-related compounds have been isolated. In many cases, these compounds have a monoterpene moiety. Biosynthesis of the teleocidin-type indole alkaloids and enzymatic reactions of teleocidin B biosynthesis are summarized in the reviews [212,213,214]. More recent investigation of *Streptomyces* sp. CNQ766 led to the identification of an unusual meroterpenoid azamerone (**297**), which has an unprecedented chloropyranophthalazinone core with a 3-chloro-6-hydroxy-2,2,6-trimethylcyclohexylmethyl side chain [215]. Along with known bacterial metabolites WS-9659A14 (lavanducyanin, **304**) and the C-2 chlorinated analog WS-9659B14 (**305**), marinocyanins A–F (**298**–**303**) were isolated from *Streptomyces* sp. CNS-284 and CNY-960. Marinocyanins represent first bromo-phenazinones with an *N*-isoprenoid substituent in the skeleton [216].

Farnesides A (**306**) and B (**307**), new sesquiterpene nucleosides, were isolated from *Streptomyces* sp. CNT-372 [217]. Two new geranylated phenazines, phenaziterpenes A (**308**) and B (**309**), were isolated from the fermentation broth of *S. niveus* SCSIO 3406 [210]. Subsequent genome analysis of this strain revealed the presence of a BGC encoding enzymes necessary for the biosynthesis of **292**–**295**, **308**, and **309** [218].

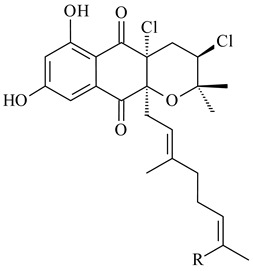

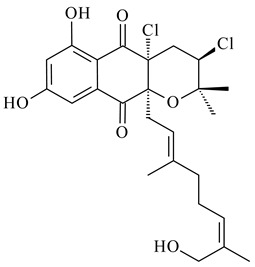

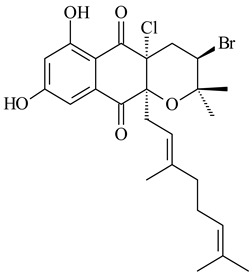
**264****265****266**
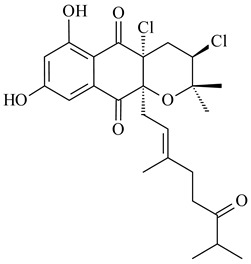

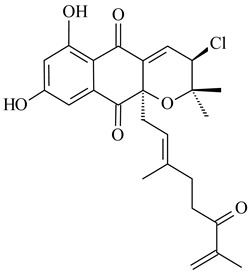

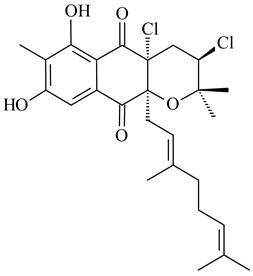
**267****268****269**
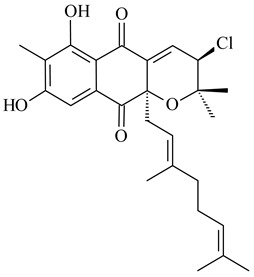

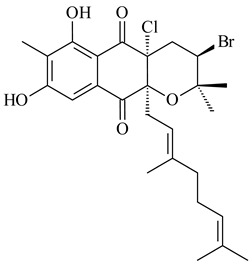

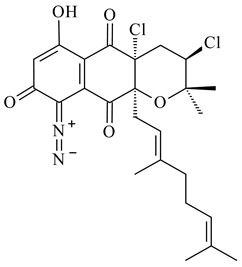
**270****271****272**
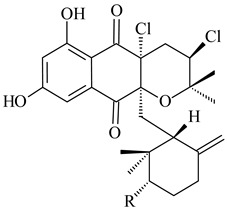

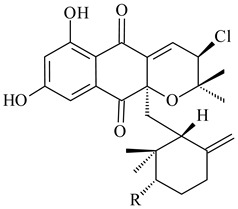

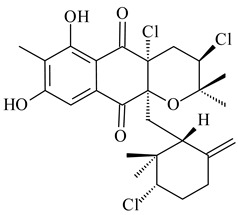
**273** R=Cl **274** R=Br**275** R=Cl **276** R=Br**277**
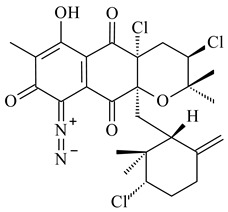

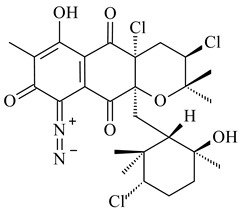

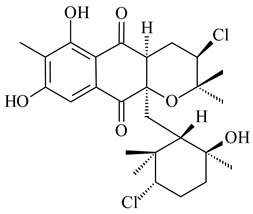
**278****279****280**
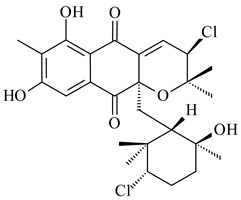

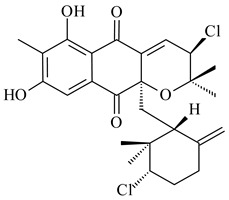

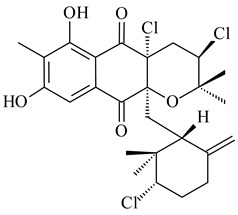
**281****282****283**
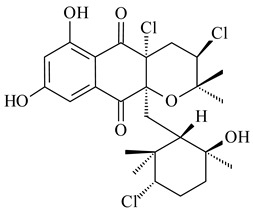

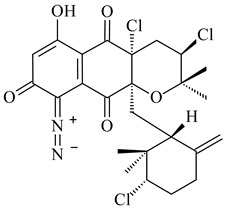

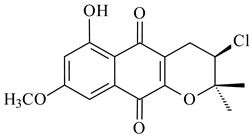
**284****285****286**
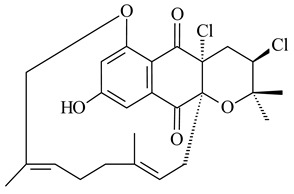

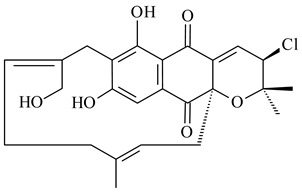
**287****288**
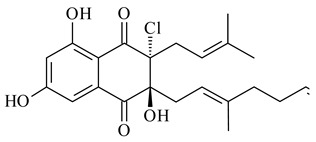

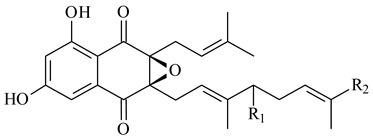
**289****290** R_1_=OH, R_2_=CH_2_OH **291** R_1_=H, R_2_=CH_3_
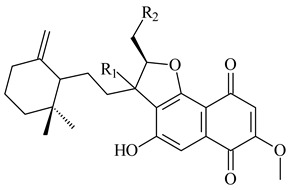

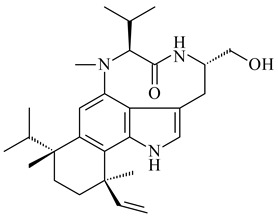

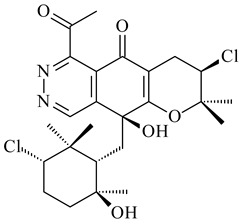
**292** R_1_=βCH_3_, R_2_=H **293** R_1_=αCH_3_, R_2_=H **294** R_1_=βCH_3_, R_2_=OH **295** R_1_=αCH_3_, R_2_=OH**296****297**
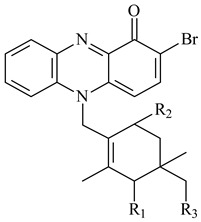

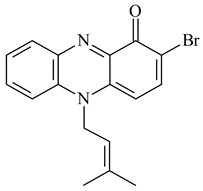

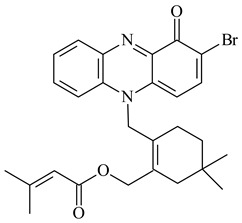
**298** R_1_=R_2_=H, R_3_=H **299** R_1_=R_2_=H, R_3_=OH **300** R_1_=OH, R_2_=H, R_3_=H **301** R_1_=H, R_2_=OH, R_3_=H**302****303**
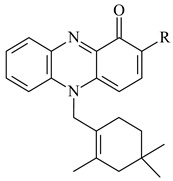

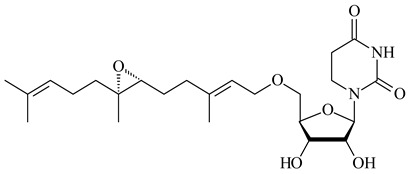
**304** R=H **305** R=Cl**306**
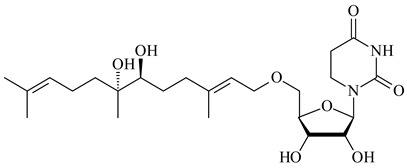
**307**


Xiamycin A (**310**) and its methyl ester (**311**) were obtained from *Streptomyces* sp. GT2002/1503 and *Streptomyces* sp. SCSIO 02999 [219,220]. Xiamycin represents one of the first examples of indolosesquiterpenes isolated from prokaryotes [221]. BGC responsible for xiamycin biosynthesis (*xia*), key enzymes and intermediates preindosespene (**314**), indosespenol (**315**), **316**, **317**, indosespene (**318**) were determined and described in [219,222,223,224] (CN102732534). Xiamycins C–E (**323**, **324**, **321**) and xiamycin B (**313**), **318**, and sespenine (**319**), along with **310**, were isolated from the culture broth of a *Streptomyces* sp. HK18 [225] and *Streptomyces* sp. HKI0595 [226], respectively. New indolosesquiterpenes oridamycins A (**326**) and B (**327**) were identified from *Streptomyces* sp. KS84 [227]. Along with **310** and oxiamycin (**320**), *Streptomyces* sp. SCSIO 02999 catalyzed the formation of dixiamycins A (**328**), B (**330**), and chloroxiamycin (**312**). Compounds **328** and **330** represent the first examples of atropoisomerism of naturally occurring *N*-*N*-coupled atropo-diastereomers [220] (CN102757908). Genome mining of *S. xinghaiensis* NRRL B-24674T resulted in the discovery of nine xiamycin analogs, including three novel compounds 19-methoxy-xiamycin (**325**), 19-carbonyl-xiamycin (**322**), and 19-hydroxy-24-methyl ester-*N*-*N*-dixiamycin (**329**) [228]. Two new compounds **331** and **332**, along with known dixiamycins (**333**–**337**, **340**), were derived from *S. olivaceus* OUCLQ19-3 [229]. Biocatalytic production of bixiamycins (**333/334**, **335**/**336**, **337**) and sulfonylbixiamycins (**338**–**340**) using *S. albus* transformant with *xia* from *Streptomyces* sp. SCSIO 02999 was patented, wherein a key role of flavin-dependent enzyme (XiaH) in biosynthesis of sulfadixiamycins, unprecedented sulfonyl-bridged alkaloid dimers, was proved [230,231] (WO2014029498)**.**

The strain *Streptomyces* sp. K04-0144, representing a novel species *S. cyslabdanicus* (=NBRC 110081T, DSM 42135T) [232], catalyzed the formation of the *N,S*-containing labdane diterpenoid cyslabdan A (**341**) and its 18-hydroxy- (cyslabdan B, **342**) and 1’-methoxy- (cyslabdan C, **343**) derivatives [233]. Genome-wide analysis of *S. cyslabdanicus* K04-0144 revealed the *cld* cluster consisting of the *cldA*, *cldB*, *cldC*, and *cldD* genes responsible for cyslabdan biosynthesis. The transformants of *S. avermitilis* SUKA22 containing the *cld* cluster produced **341** as well as its new 17-hydroxy- (**344**) and 2α-hydroxy- (**345**) derivatives, and (7*S*,8*S*,12*E*)-8,17-epoxy-7-hydroxylabda-12,14-diene (**346**). Insertion of the *cld*-like *rmn* cluster from *S. anulatus* GM95 in *S. avermitilis* SUKA22 resulted in raimonol (**171**) [149]. In addition, the heterologous expression of the *lrdABDC* cluster from *S. thermocarboxydus* K155 in the *S. cyslabdanicus* K04-0144*Δcld* mutant led to the formation of **341** and **346** [147].

*Streptomyces* sp. KO-3988 [234], *S. griseus* CB00830 [235], and *Streptomyces* sp. SN194 [152] synthesized novel oxaloterpins A–E (**347**–**351**). Two new *Cl*-containing diterpenoids chloroxaloterpins A (**352**) and B (**353**) containing unique groups [(2-chlorophenyl)amino]carbonyl and 2-[(2-chlorophenyl)amino]-2-oxo-acetyl, respectively, were identified among the metabolites of *Streptomyces* sp. SN194 [152].

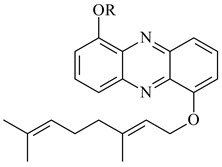

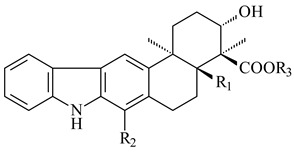

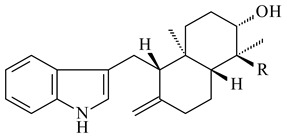
**308** R=H **309** R=CH_3_**310** R_1_=R_2_=R_3_=H **311** R_1_=R_2_=H, R_3_=CH_3_ **312** R_1_=H, R_2_=Cl, R_3_=H **313** R_1_=OH, R_2_=R_3_=H**314** R=H **315** R=CH_2_OH **316** R=CH(OH)_2_ **317** R=CHO **318** R=COOH
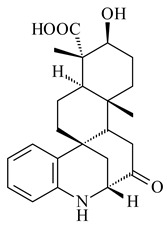

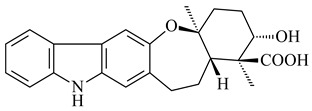

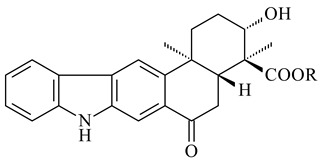
**319****320****321** R=CH_3_ **322** R=H
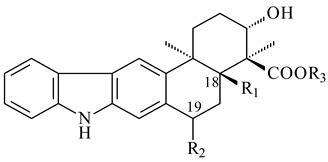

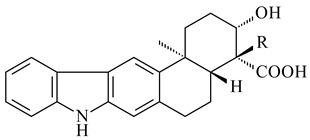
**323** R_1_=H, R_2_=αOH, R_3_=H **324** R_1_=H, R_2_=αOH, R_3_=CH_3_ **325** R_1_=H, R_2_=OCH_3_, R_3_=H**326** R=CH_3_ **327** R=CH_2_OH
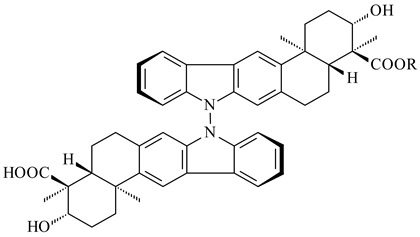

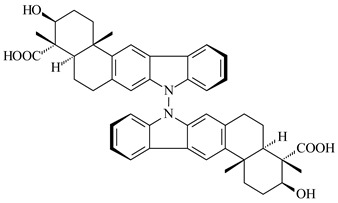
**328** R=H **329** R=CH_3_**330** dixiamycin B
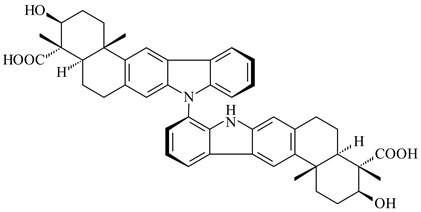

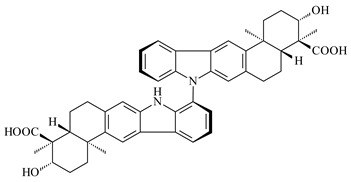
**331****332**
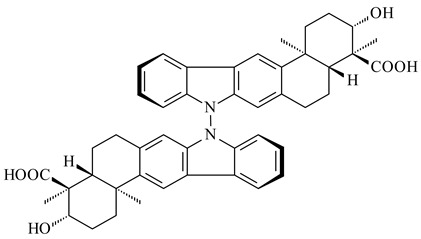

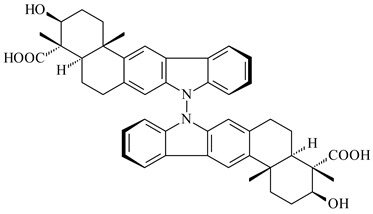
**333****334**
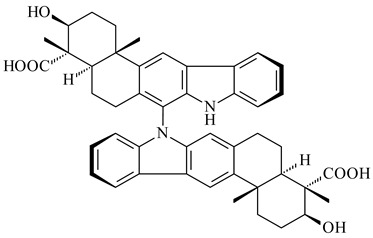

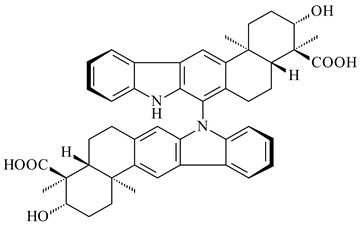
**335****336**
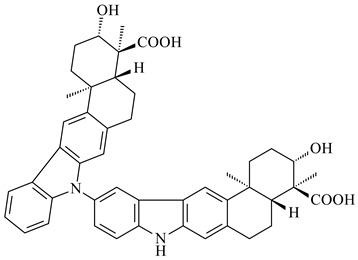

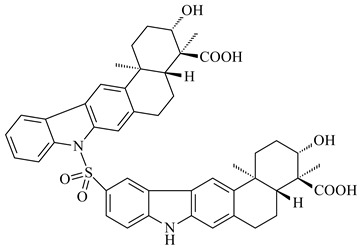
**337****338**
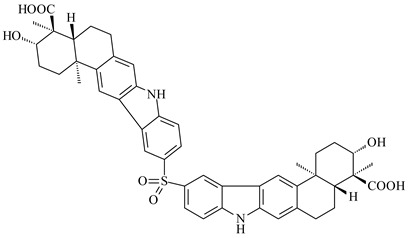

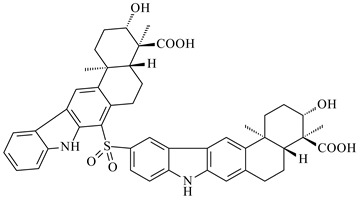
**339****340**
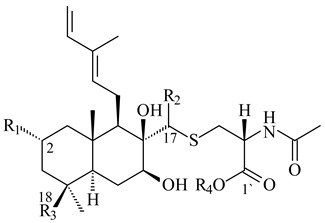

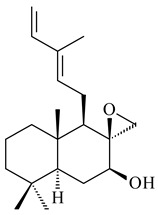
**341** R_1_=R_2_=H, R_3_=CH_3_, R_4_=H **342** R_1_=R_2_=H, R_3_=CH_2_OH, R_4_=H **343** R_1_=R_2_=H, R_3_=R_4_=CH_3_
**344** R_1_=H, R_2_=OH, R_3_=CH_3_, R_4_=H **345** R_1_=OH, R_2_=H, R_3_=CH_3_, R_4_=H**346**



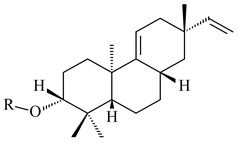



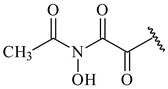



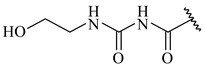



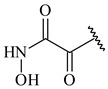


**347** R= **348** R=**349** R=

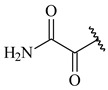

**351** R=NH_2_

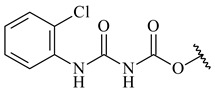



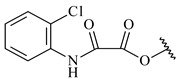

**350** R=
**352** R=**353** R=

*Streptomyces* sp. Tü6071 produced phenalinolactones A–D (**354**–**357**), tricyclic terpene glycosides, and their derivatives **359**–**362**, **365**, and **366** [236,237]. The mutants of *Streptomyces* sp. Tü6071 with inactivated oxygenase genes (*plaO2*, *plaO3*, *plaO5*), dehydrogenase genes (*plaU*, *plaZ*) and putative acetyltransferase gene (*plaV*) yielded phenalinolactone derivatives PL HS2 (**364**), PL X1 (**363**) PL HS6 (**367**), and PL HS7 (**368**) [238]. Later, the intermediates of synthesis of phenalinolactones A (**354**) and D (**357**) were identified as PL IM1 (**370**) and PL IM2 (**369**), respectively [239]. Heterologous expression of the phenalinolactone BGC (35 genes) in *S. coelicolor* M512 resulted in the formation of the non-glycosylated derivative phenalinolactone E (**358**) [240]. 

Tiancilactones A–K (**371**–**381**), close structural analogues of phenalinolactones, were discovered by genome mining of diterpene synthases in *Streptomyces* sp. CB03234 and *Streptomyces* sp. CB03238. Tiancilactones are characterized by a highly functionalized diterpene backbone, which comprises chloroanthranilate and γ-butyrolactone moieties, and exhibit antibacterial activity [241]. Two new terpenoids with unique a 6-6-6-fused ring system and an oxidized unsaturated *γ*-lactone, namely trinulactones A (**382**) and B (**383**), were isolated from *Streptomyces* sp. S006 [242].

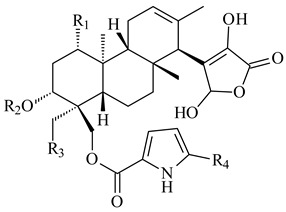

R_1_R_2_R_3_R_4_**354**OH-CO-CH_3_
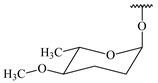
CH_3_**355**OH-CO-CH_3_
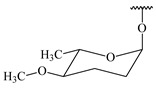
H**356**OH-CO-CH_3_
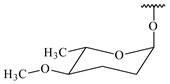
-CH_2_-O-CH_3_**357**H-CO-CH_3_
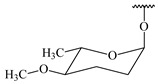
CH_3_**358**H-CO-CH_3_OHCH_3_**359**H-CO-CH_3_
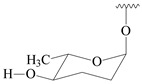
CH_3_**360**HHHCH_3_**361**H-CO-CH_3_HCH_3_**362**HHOHCH_3_**363**HH
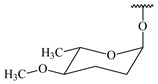
CH_3_**364**H-CO-CH_3_
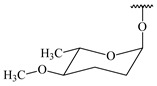
-CH_2_-O-CH_3_
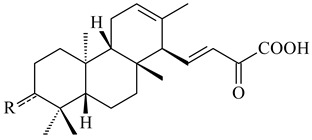

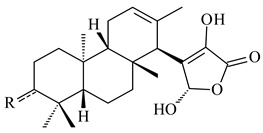
**367** R=αOH**368** R=O
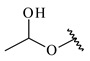
**365** R=αOH **366** R=O
**369** R
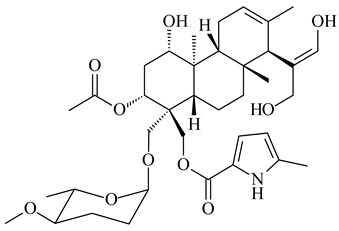

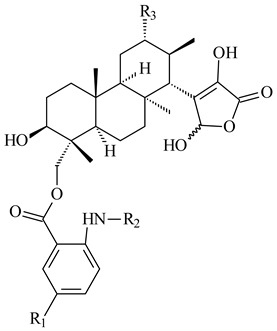
**370****371** R_1_=Cl, R_2_=CH_3_, R_3_=OCH_3_ **372** R_1_=H, R_2_=CH_3_, R_3_=OCH_3_ **373** R_1_=Cl, R_2_=CH_3_, R_3_=OH **374** R_1_=Cl, R_2_=CH_3_, R_3_=oxo **375** R_1_=Cl, R_2_=H, R_3_=OCH_3_
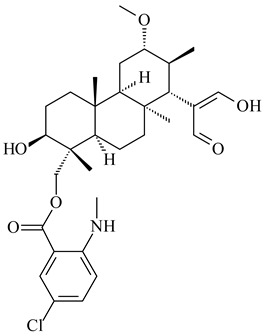

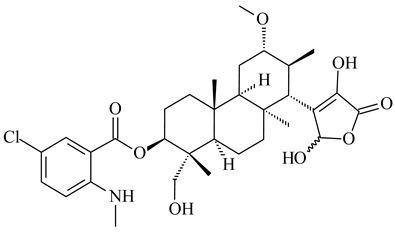
**376****377**
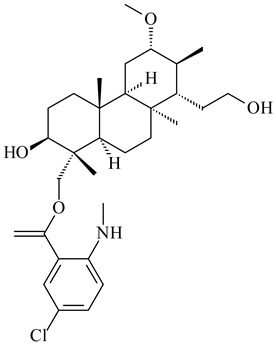

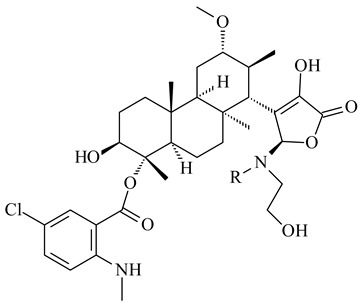
**378****379** R=H **380** R=CH_3_
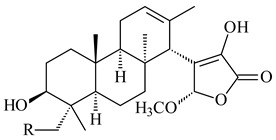

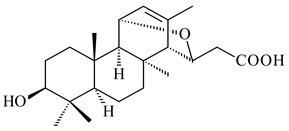
**381** R=H **382** R=OH**383**

Fusicomycin A (**384**), its isomer **385**, and fusicomycin B (**386**) were separated from the fermentation broth of *S. violascens* YIM 100212 [164]. Two new non-cytotoxic diterpene streptooctatins A (**387**) and B (**388**) were obtained from *Streptomyces* sp. KCB17JA11 [243]. Actinoranone (**389**) is new meroterpenoid derived from *Streptomyces* sp. CNQ-027 consisting of an unprecedented dihydronaphthalenone polyketide linked to a bicyclic diterpenoid [244].

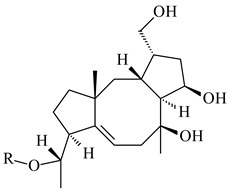

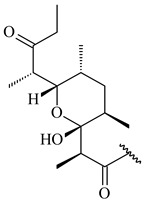

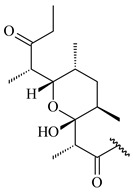

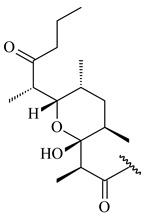

**384** R=**385** R=**386** R=
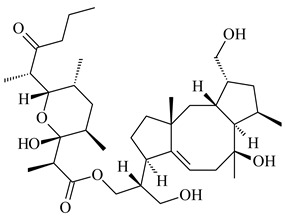

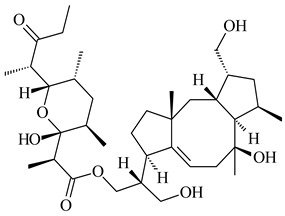

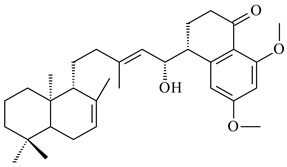
**387****388****389**

*S. platensis* MA7327 and *S. platensis* MA7339 were shown to synthesize platensimycin (**390**) and platencin (**391**), representatives of a new class of broad-spectrum antibiotics against Gram-positive bacteria, in particular *S. aureus* [245,246]. Further study proved the involvement of *ent*-kaurene and *ent*-atiserene synthases in biosynthesis of **390** and **391**, representing a new biosynthetic pathway for diterpenoids [247,248,249]. The crystal structure of PtmT2, an *ent*-copalyl diphosphate synthase involved in the biosynthesis of **390** and **391** in *S. platensis* CB00739, was described. PtmT2 catalyzed the cyclization of GGPP to *ent*-CPP, which subsequently channeled into (16*R*)-*ent*-kauran-16-ol (**392**) or *ent*-atiserene (**393**) by two distinct type (canonical or UbiA-type) diterpene synthases specific for biosynthesis of **390** or **391**, respectively [250]. The metabolically engineered strains *S*. *platensis* SB12002 and SB12600 produced **390** and **391** with yields of 323 ± 29 mg/L and 255 ± 30 mg/L, respectively, hundreds of times greater than those of wild-type strains [251,252] (US20090081673). *S. platensis* SB12600, in addition to **391**, accumulated eight new congeners, platencins A2–A9 (**394**–**402**) [253]. A method for obtaining **390** using the mixed culture of *S. hygroscopicus* HOK021 (NITE P-02560) and *Tsukamurella pulmonis* TP-B0596 was patented (JP2019149945). Exemplified by **390** and **391**, a method of searching for novel natural compounds based on the analysis of biosynthetic genes was proposed (WO2015200501). Data on the biosynthesis features and biological activity of natural and synthetic analogues of platensimycin and platencin were summarized in the reviews [254,255].

The intermediates of hopanoids biosynthesis, *N*-containing aminobacteriohopanetriol (**403**), and adenosylhopane (**405**), as well as bacteriohopanetetrol (**404**) and ribosylhopane (**406**), were determined. *Orf14* and *orf18* of *S. coelicolor* A(3)2 responsible for the synthesis of **403** were identified [176]. 

Among the secondary metabolites of *Streptomyces* sp. YIM 56130, triterpene glycoside soyasaponin I (**407**) [94] with a wide spectrum of biological activities [256] was obtained. The tetraterpene glycoside KS-505a (longestin, **408**) produced by *S. argenteolus* A-2 (FERM BP2065) has a unique structure consisting of a tetraterpene skeleton with 2-*O*-methylglucuronic acid and *O*-succinyl benzoate moieties [257].

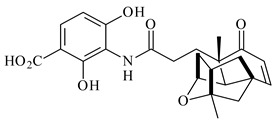

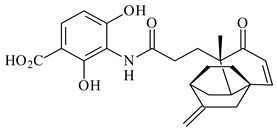

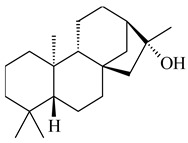
**390****391****392**
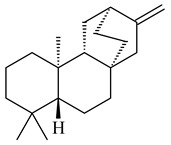

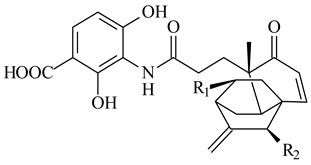
**393****394** R_1_=OH, R_2_=H **395** R_1_=H, R_2_=OH
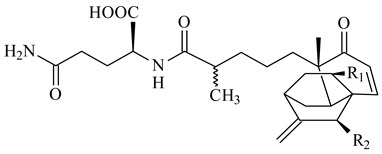

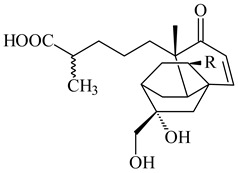
**396** R_1_= R_2_=H **397** R_1_=OH, R_2_=H **398** R_1_=H, R_2_=OH**399** R=OH
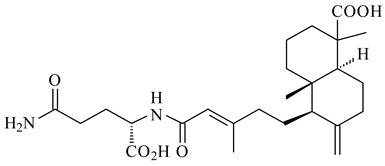

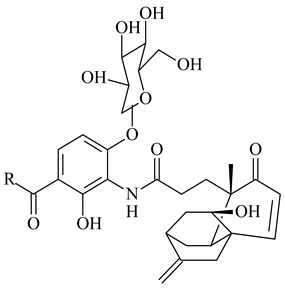
**400****401** R=SCH_3_ **402** R=OCH_3_
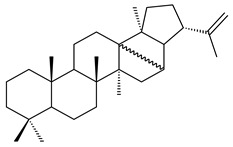

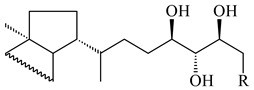

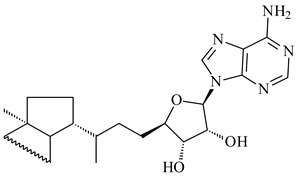
**403** R=NH_2_ **404** R=OH**405**
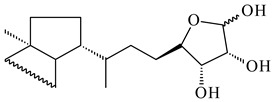

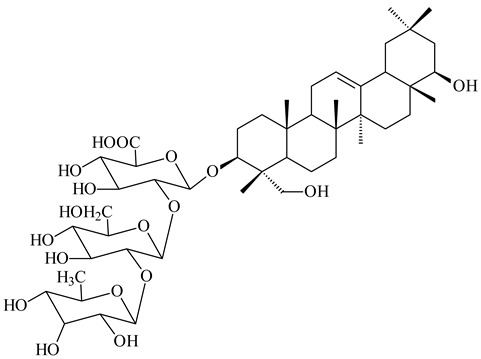
**406**


**407**
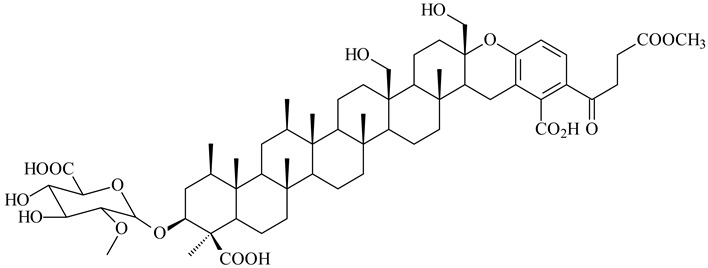
**408**

### 2.2. Terpene Derivatives Produced by Others Actinomycetes and Their Enzymes

Although most of the found actinomycete terpene derivatives are synthesized by streptomycetes, there is an increasing number of publications on terpene biosynthesis by representatives of the genera *Nocardiopsis*, *Amycolatopsis*, *Isoptericola*, *Saccharopolyspora*, *Salinispora*, *Kitasatosporia*, *Verrucosispora*, etc. The compounds produced are represented mainly by sesqui- and diterpenes and their derivatives.

#### 2.2.1. Mono- and Sesquiterpenes and Their Derivatives

Among the secondary metabolites of *Nocardiopsis chromogenes* YIM 90109, two new monocyclic germacradiene-type sesquiterpenoids germacradiene-9β,11-diol (**409**) and 11-hydroxy-germacradien-2-one (2-oxygermacradienol, **410**) were identified along with the known geosmin-type sesquiterpenoid **46** [258]. The TSs from *Kitasatospora setae* KM-6054 [259] and *Micromonospora marina* DSM 45555 [260] catalyzed the formation of hedycaryol (**411**) and (−)-germacrene A (**27**), respectively. The ability to produce bicyclic 2-methylisoborneol (**6**) and geosmin (**22**) was described for *Nocardia cummidelens* and *N*. *fluminea* [59]. The transformant of *S. avermitilis* carrying the genes from *Saccharopolyspora erythraea* NRRL2338 yielded 2-methylisoborneol (**6**), while *Micromonospora olivasterospora* KY11048 synthesized 2-methyleneornane (**412**) [58].

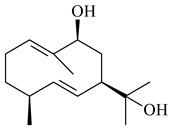

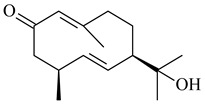

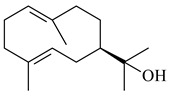

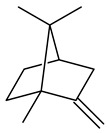
**409****410****411****412**

Two new monocyclic sesquiterpenoids (**413** and **414**) were isolated from the culture medium of *Amycolatopsis alba* DSM 44262 [261]. Among the secondary metabolites of *Isoptericola chiayiensis* BCRC 16888, a new sesquiterpenoid isopterchiayione (**415**) was registered [262]. A new trichoacorenol sesquiterpene synthase from *Amycolatopsis benzoatilytica* DSM 43387 catalyzing the formation of a bicyclic sesquiterpenoid (**416**) was described [263]. *Verrucosispora gifhornensis* YM28-088 [264] and *Verrucosispora* sp. FIM06031 produced bicyclic sesquiterpenoid cyperusol C (**417**) and FW03104 (**418**) (CN101898936), respectively. 

Terpene synthases from *Streptosporangium roseum* DSM 43021 and *Kitasatosporia setae* KM-6054 afforded tricyclic sesquiterpenoids *epi*-cubebol (**419**) [265] and new corvol ethers A (**420**) and B (**421**) [265,266], respectively. The terpene synthase from *Saccharothrix espanaensis* DSM 44229 [103] was incubated with FPP to yield a sesquiterpene (*E*)-β-caryophyllene (**93**).

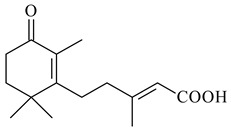

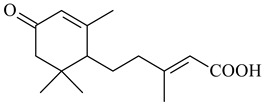

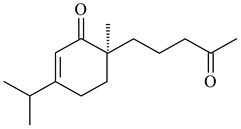
**413****414****415**
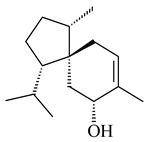

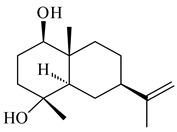

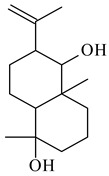
**416****417****418**
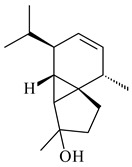

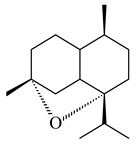

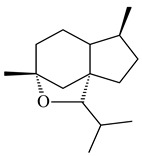
**419****420****421**

#### 2.2.2. Di- and Triterpenes and Their Derivatives

The TS from *Micromonospora marina* DSM 45555 was functionally characterized to produce micromonocyclol (**422**)**,** a new diterpene alcohol with a rare 15-membered ring [267]. *Mycobacterium tuberculosis* H37Rvн synthesized unique bicyclic diterpenoids, which presumably block the formation of phagolysosomes in human macrophages. The *Rv3377c* and *Rv3378c* genes proved to be responsible for synthesis of tuberculosinol (5(6),13(14)-halimadiene-15-ol, **423**), 13*R*- (**424**) and 13*S*-isotuberculosinol (5(6),14(15)-halimadiene-13-ol, **425**), and nosyberkol (**426**) (previously identified as edaxadiene). The analogs of *Rv3377c* and *Rv3378c* were found in the virulent strains of *M. tuberculosis* CDC1551 and *M. bovis* subsp. *bovis* AF2122/97, but did not occur in non-pathogenic strains [268,269,270,271,272]. Later, the crystal structure of the Rv3377 diterpene synthase was described [273].

A bicyclic terpenoid terpentecin (**427**) was firstly separated from the fermentation broth of *Kitasatosporia griseola* MF730-N6 (syn. *Streptomyces griseolosporeus* MF730-N6) in 1985 [274]. A BGC responsible for the terpentecin biosynthesis includes seven ORFs (ORF8-ORF14). Expression of two cyclase genes *ORF11* and *ORF12* in *S. lividans* together with the GGDP synthase gene resulted in the formation of a new cyclic diterpene *ent*-clerod-3,13(16),14-triene (terpentetriene, **428**) with a structure similar to **427** [275,276,277]. CYC2, which converted terpentedienyl phosphate (**429**) to **428**, accepted labdane-type diterpene diphosphates (+)-CDP (**430**), *syn*-CDP (**431**), (−)-*ent*-CDP (**432**), as well as halimane-type diterpene diphosphate (TBPP, **433**) and catalyzed the formation of corresponding derivatives (**434**–**437**) [278].

Heterologous expression of the biosynthetic *terp1* operon from *Salinispora arenicola* CNS-205 in *E. coli* led to the generation of isopimara-8,15-dien-19-ol (**438**). It should be noted that this terpenoid was not observed in pure cultures of *S. arenicola* CNS-205. Apparently, the *terp1* operon was expressed under certain conditions, for example, in the presence of other marine organisms [279]. The terpene synthase Sat1646 from *Salinispora* sp. PKU-MA00418 accepted CPP and *syn*-CPP and produced *syn*-isopimaradiene/pimaradiene analogues (**180**, **439**–**446**). Compound **439** possess a unique and previously unreported 6-6-7 ring skeleton [150]. New hydroxylated derivatives of isopimaradiene, gifhornenolones A (**447**) and B (**448**), were isolated from the culture medium of *Verrucosispora gifhornenensis* YM28-088 [264]. Among secondary metabolites of *Micromonospora haikouensis* G039 [280] and *Microbispora hainanensis* CSR-4 [281], new diterpenoids isopimara-2-one-3-ol-8,15-diene (**449**) and 2α-hydroxy-8(14),15-pimaradien-17,18-dioic acid (**450**) were identified, respectively.

*Actinomadura* sp. SpB081030SC-15 [282] and *Actinomadura* sp. KC 191 [283] synthesized new JBIR-65 (**451**) and actinomadurol (**452**), rare bacterial C-19 norditerpenoids. A norditerpenoid k4610422 (**453**), originally discovered from a mesophilic rare actinomycete of the genus *Streptosporangium*, was isolated from the culture extract of a thermophilic actinomycete *Actinomadura* sp. AMW41E2 [284].

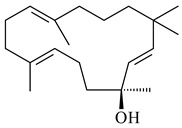

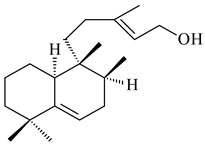

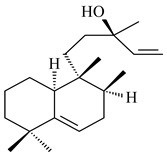

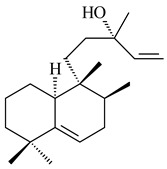
**422****423****424****425**
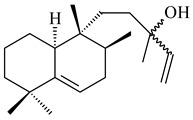

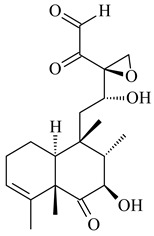

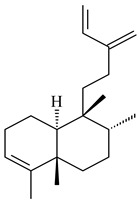

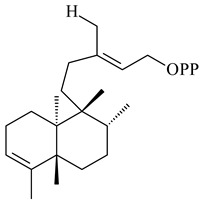
**426****427****428****429**
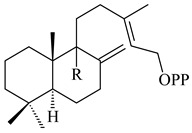

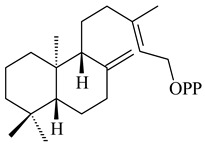

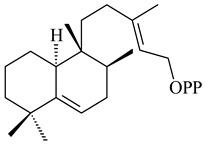

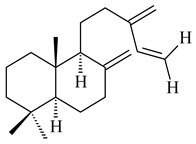
**430** R=αH **431** R=βH**432****433****434**
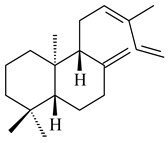

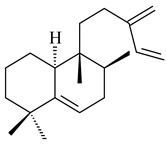

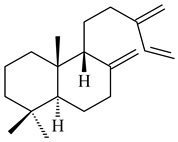

**435****436****437**

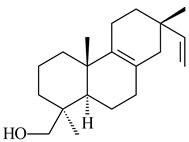

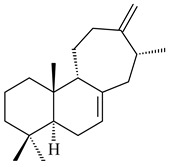

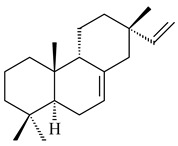

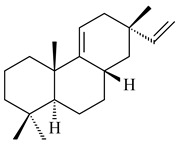
**438****439****440****441**
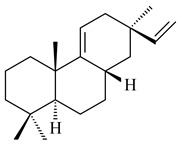

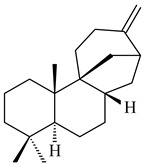

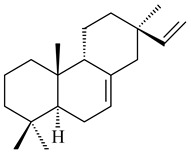

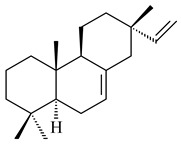
**442****443****444****445**
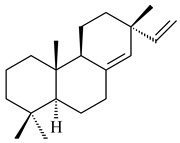

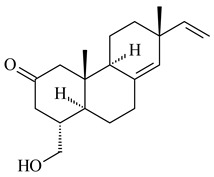

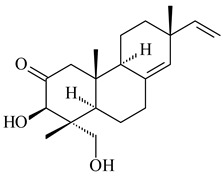

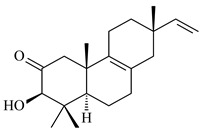
**446****447****448****449**
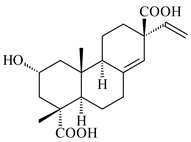

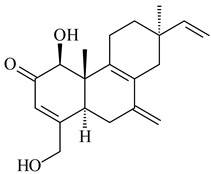

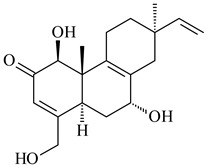

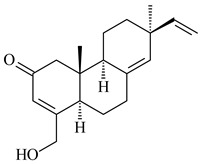
**450****451****452****453**

Diterpene synthases from *Catenulispora acidiphila* DSM 44928 and *Saccharopolyspora spinosa* NRRL 18395 produced new di- and tricyclic catenul-14-en-6-ol (**454**), isocatenula-2,14-diene (**455**), isocatenula-2(6),14-diene (**456**) [285], and spinodienes A (**457**), B (**458**), and 2,7,18-dolabellatriene (**459**) [286], respectively. All obtained compounds are characterized by unique carbon skeletons.

Terpene synthases isolated from *Nocardia testacea* NBRC 100365 and *N. rhamnosiphila* NBRC 108938 accepted GGPP, but not GPP, FPP, or GFPP as a substrate, which was converted by both enzymes in a tetracyclic diterpene phomopsene (**460**) [169]. *Allokutzneria albata* DSM 44149 encoded four diterpene synthases that catalyze the formation of mono-, tri-, and tetracyclic compounds: new spiroalbatene (**461**), bonnadiene (**462**) and allokutznerene (**463**), and known compounds: cembrene A (**164**), thunbergol (**464**), phomopsene (**460**), and spiroviolene (**200**) [287,288].

Hopanoid lipids (**465**–**482**) were found in the genus *Frankia* [289] with the highest level among all known organisms. Short stretches of DNA have been identified that are thought to contain squalene-hopene cyclase genes (shc) [290]. A new sesquarterpenoid identified as heptaprenylcycline B (**483**) was isolated from the cell walls of nonpathogenic mycobacteria [291,292].

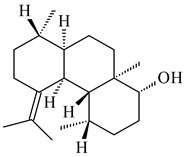

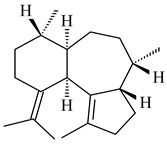

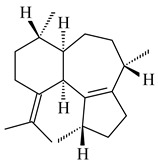

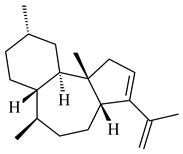
**454****455****456****457**
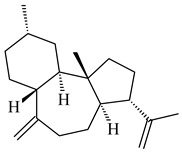

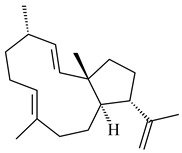

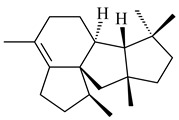

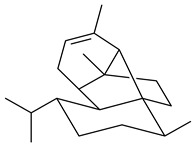
**458****459****460****461**
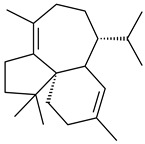

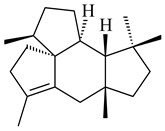

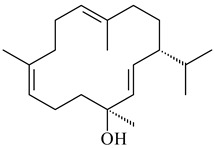

**462****463****464**




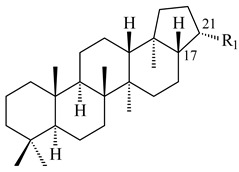



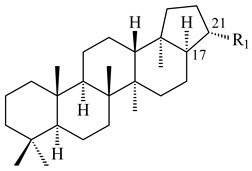



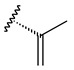



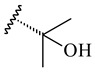



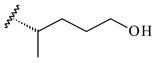



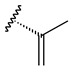



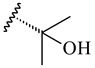



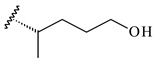

**465** R_1_**466** R_1_**467** R_1_**472** R_1_**473** R_1_**474** R_1_**468** R_1_ 
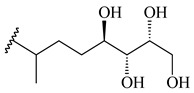
**475** R_1_ 
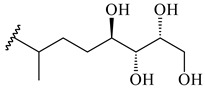
R_1_ 
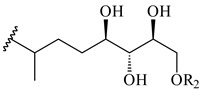
**469** R_2_=H **470** R_2_=-COCH_2_CH_3_ **471** R_2_=-COCH_2_C_6_H_5_R_1_ 
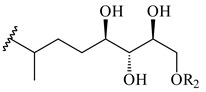
**476** R_2_=H **477** R_2_=-COCH_2_CH_3_ **478** R_2_=-COCH_2_C_6_H_5_

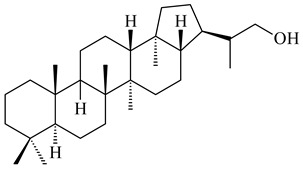



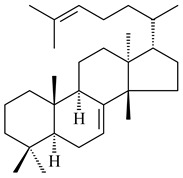



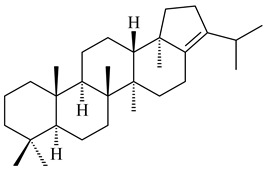



**479**

**480**

**481**



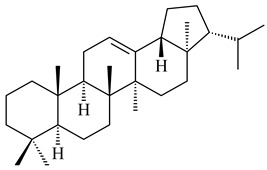



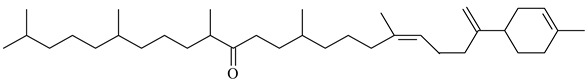



**482**

**483**



#### 2.2.3. Hybrid Metabolites (Meroterpenoids)

*Verrucosispora* sp. FIM06031 synthesized bicyclic sesquiterpenoid FW03105 (**484**) (CN101921721). *Saccharomonospora* sp. CNQ-490 produced saccharoquinoline (**485**), meroterpenoid with drimane-type sesquiterpene unit [293]. Two new halimane-type diterpenoids, micromonohalimanes A (**486**) and B (**487**), were derived from *Micromonospora* sp. WMMC-218, a symbiont of marine ascidians *Symplegma brakenhielmi* [294]. Further research of Rv3378c from *Mycobacterium tuberculosis* H37Rvн revealed that this enzyme catalyzed the formation of 1-tuberculosinyladenosine (**488**) and its two isomers, one of which was identified as *N^6^*-tuberculosinyladenosine (**489**). Compounds **488** and **489** are specific diterpene nucleosides of pathogen of *Mycobacterium tuberculosis* and can serve as chemical markers of infection [295,296,297]. Heterologous expression of gene pair *Rv3377c*-*Rv3378c* from *M. tuberculosis* H37Rvн in *M. kansasii* led to the production of 1-tuberculosinyladenosine (**488**) [298].

The ability of *Nocardia brasiliensis* IFM 0406 (now *N. terpenica*) to synthesize diterpene glycoside brasilicardin A (**490**) was first described in 1999 [299]. Brasilicardin A (**490**) displays a unique structure consisting of a diterpene skeleton with *L*-rhamnose, *N*-acetylglucosamine, amino acid, and 3-hydroxybenzoate components [300]. Later, three new terpenoids were derived from *N. terpenica* IFM0406 and identified as brasilicardins B–D (**491**–**493**) [301]. The heterologous expression of a biosynthetic cluster (*bra0-12*), responsible for the synthesis of **490**, in *Amycolatopsis japonicum* (*A. japonicum*::bcaAB01) led to the formation of four brasilicardin congeners, namely BraC (**492**), BraD (**493**), BraC-agl (BraE, **494**), and BraD-agl (BraF, **495**) [302,303,304,305]. The use of the *S. griseus*::bcaAB01 (pRHAMO) transformant containing the biosynthetic cluster of brasilicardin A and a plasmid with a biosynthetic cassette for the generation of TDP-*L*-rhamnose resulted in increased yields of compounds **492** (1669 mg/L), **495** (926 mg/L), and a new metabolite (**496**) (15 mg/L). The target **490** was obtained through a five-step chemical modification of **494** [306].

Cloning and activation of the atolypene (*ato*) gene cluster from *Amycolatopsis tolypomycina* NRRL B-24205 in *S. albus* led to the characterization of two unprecedented tricyclic sesterterpenoids atolypenes A (**497**) and B (**498**) [307]. Terretonin N (**499**), a new highly oxygenated unique tetracyclic 6-hydroxymeroterpenoid, was derived from *Nocardiopsis* sp. LGO5 [308].

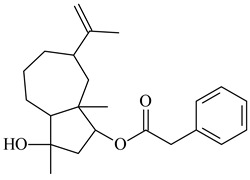

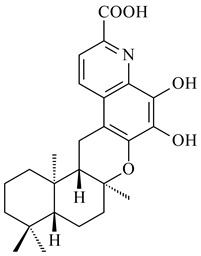

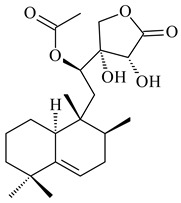

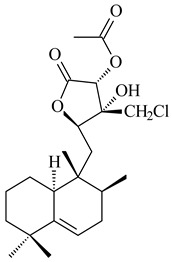
**484****485****486****487**
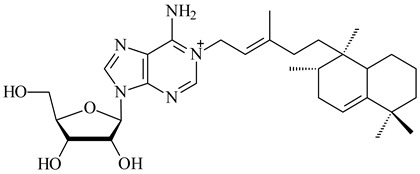

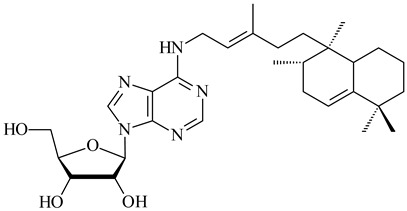
**488****489**
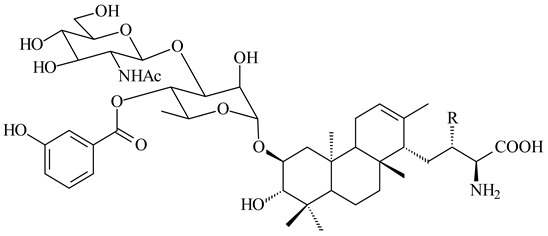
**490** R=OCH_3_**491** R=H
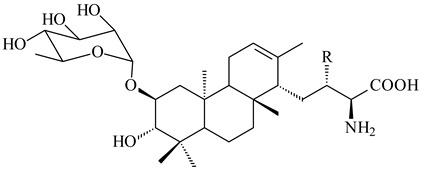

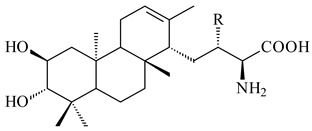
**492** R=OCH_3_ **493** R=H**494** R=OCH_3_ **495** R=H
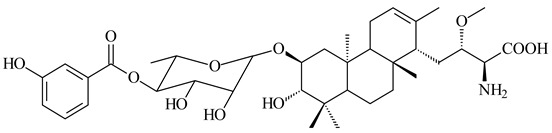
**496**
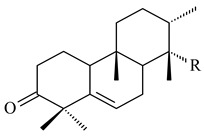

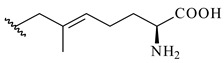

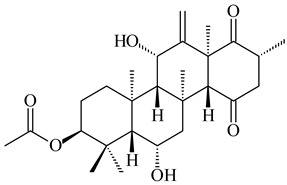
**499****497** R
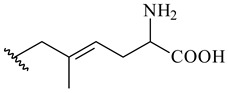
**498** R

## 3. Discussion

**Figure 3:**
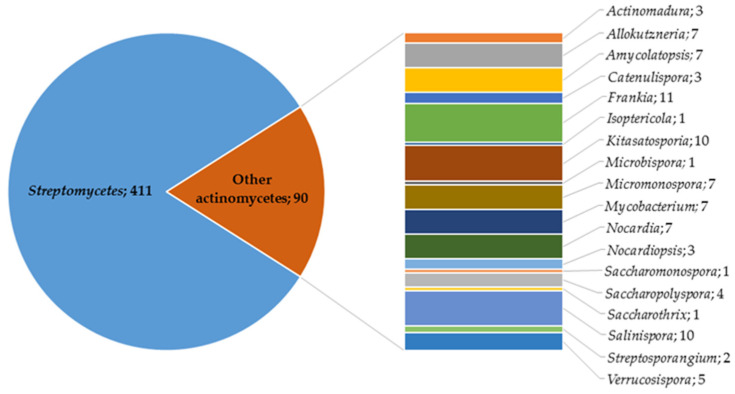
The total number of identified terpene derivatives (mentioned in the review) produced by different genera of actinomycetes.

**Figure 4:**
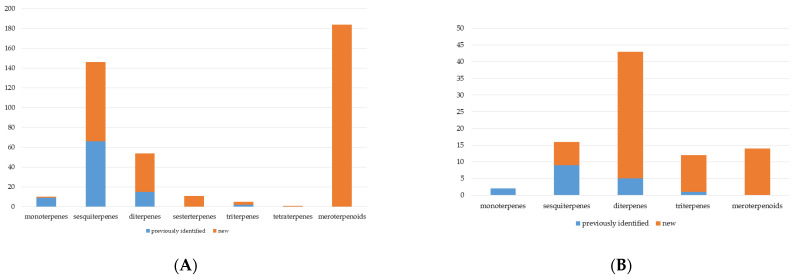
Various groups of terpene derivatives synthesized by actinomycetes: (**A**) the genus *Streptomyces*, (**B**) other genera.

The present review demonstrates that actinomycetes synthesize a wide variety of terpene derivatives ranging from monocyclic monoterpenes to polycyclic tri- and tetraterpenes and their various derivatives. Most actinomycete terpene derivatives are produced by *Streptomyces*, however, terpene biosynthesis by *Allokutzneria*, *Amycolatopsis*, *Frankia*, *Kitasatosporia*, *Nocardia*, *Salinispora*, *Verrucosispora*, etc., have been recently reported (Figure 3). The total number of identified terpenes and their derivatives exceeds 500. Among terpenes and terpenoids, sesqui- and diterpenoids predominate. The ability of streptomycetes to synthesize a wide range of hybrid metabolites (meroterpenoids), the total number of which exceeds 190, was shown. More than 350 actinomycete-derived terpenoids and meroterpenoids are novel compounds and frequently with unique carbon skeletons (Figure 4). 

An extensive development of genome-sequencing technologies and bioinformatics tools have allowed the discovery of BCGs (including silent ones) in the genome of actinomycetes. That terpenoids and meroterpenoids are predominantly found among *Streptomyces* strains is presumably due to plenty of available genetic information about this group of actinomycetes. As of 26 June 2022, 1784 scaffold-level and 745 complete-level genome sequences of *Streptomyces* strains were available in the NCBI database. Recent genetic studies have shown that the biosynthetic potential of these actinomycetes is enormous. A genome-wide analysis of 22 *Streptomyces* species revealed more than 900 biosynthetic clusters; for most of these, the products are still unidentified [309]. In addition, *Streptomyces* are preferred hosts for the heterologous expression of terpene biosynthetic clusters from other microorganisms [48,50,310]. Since 2015, high biosynthetic potential of actinomycete genera such as *Saccharopolyspora* [311], *Nocardiopsis* [312], *Rhodococcus* [313,314], *Salinispora* [315], *Verrucosispora* [316], and *Actinomadura* [317] have been demonstrated. For instance, a genome-wide analysis of terpentecin- or brasilicardin-producing strains *K. griseola* MF730-N6 [318] and *N. terpenica* IFM0406 [319] revealed 15 and 47 BGCs yielding unidentified natural products, respectively. One of the main problems in terpene biosynthesis is that most biosynthetic clusters are silent; therefore, searching for methods of their activation is an urgent research direction. Currently, great success has been achieved in this field due to methods of heterologous expression and/or genome editing of the native producer [320]. Genomic data of the described actinomycete species demonstrated that 90% of the biosynthetic potential of these microorganisms is untapped yet and the possibility of discovering novel terpenoids with potential therapeutic effects remains [15,52,310,321]. Microbial collections can serve as a “springboard” for the discovery and patenting of new producers of bioactive terpene derivatives, as they include identified and well-characterized pure microbial cultures. For instance, the Regional Specialized Collection of Alkanotrophic Microorganisms (acronym IEGM, Perm, Russia; World Federation for Culture Collections # 285; USU 73559; http://www.iegmcol.ru/strains, accessed on 25 March 2022) contains more than 3000 strains of actinomycetes with a wide range of metabolic capabilities, which are promising for biocatalytic production of terpene derivatives [322,323,324,325,326] (RU0002529365).

Unlike the biosynthesis of well-studied secondary metabolites, such as polyketides and nonribosomal peptides, the prediction of terpene structures requires detailed understanding of the cyclization mechanisms and the structural characteristics of bacterial TSs [321,327]. In this regard, a separate research area is isolation of individual actinomycete terpene synthases, and description of their structural and mechanistic characteristics, as well as the study of terpene cyclization mechanisms. The crystal structures of linalool/nerolidol, 2-methylisoborneol, germacradienol/germacrene D, selina-4(15),7(11)-diene, *epi*-zizaene, pentalenene, cucumene, (*E*)-biformene synthases, and other TSs isolated from streptomycetes were characterized. In turn, genome mining of streptomycetes as producers of naphthoquinone-based meroterpenoids led to the discovery of unique prenyltransferase (PTase) and vanadium-dependent haloperoxidase enzymes (VHPO) [182,183]. For instance, the high-resolution crystal structures of two homologous members of the VHPO family associated with napiradiomycin biosynthesis, NapH1 and NapH3, were characterized [184]. It has been found that bacterial TSs, PTases, and VHPOs differ significantly from the plant or fungi ones as well as from each other. Moreover, they are capable of producing dozens of different compounds, which distinguishes them from most bacterial biosynthetic enzymes [46]. By the example of an *epi*-zizaene synthase, the successful application of site-directed mutagenesis of the enzyme to control the range of the compounds produced was proved [110,122] (WO2015120431). 

**Figure 5:**
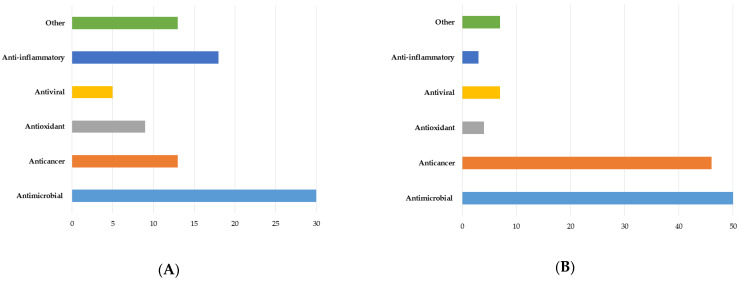
Biological activity of actinomycete terpenoids (**A**) and meroterpenoids (**B**). (The x-axis indicates the number of compounds with a certain type of activity (for meroterpenoids isomers also were counted)).

Actinomycetes produce terpenoids with various biological and pharmacological activities such as antimicrobial, anticancer, antioxidant, antiviral, anti-inflammatory, immunosuppressive, etc. (Table 2). However, the bioactivity for most of the new actinomycete-derived terpenoids has not yet been determined but may be discovered in the future. For instance, napyridymycins A1 and A80915 A, B, C, D were originally known as antimicrobial agents, but after 2010, their high antiviral and cytotoxic activity have been determined. Among the biologically active actinomycete terpenoids, compounds with pronounced antimicrobial activity predominate (Figure 5A). They seem to inhibit the growth of extraneous microflora and render actinomycetes competitive in the microbial community. This statement is confirmed by the fact that some actinomycetes begin to produce terpenoids in the presence of other microorganisms. Thus, *S. cinnabarinus* PK209 and *S. hygroscopicus* HOK021 (NITE P-02560) synthesize the diterpene lobocompactol and the antibiotic platensimycin in the presence of the Gram-negative *Alteromonas* sp. KNS-16 [140] and the Gram-positive *Tsukamurella pulmonis* TP-B0596 (JP2019149945), respectively. The effectiveness of actinomycete terpenoids and meroterpenoids, namely pentalenolactone, albaflavenone, platensimycin, platencin, terpentecin, lavanducyanin, marinocyanins A–C, furaquinocin L, 3-dechloro-3-bromonapyradiomycin A1, napyradiomycin A1, and merochlorin A, as promising antibiotics has been proven. This is true for cyslabdan, which enhances the action (1000-fold) of the antibiotic imipenem against MRSA. In addition to high antibacterial activity, many meroterpenoids, such as napyradiomycins B1, B3, B4, A80915A, B, C, furaquinocins A and B, murayaquinone, marinocyanin A–C, and saccharoquinoline, exhibit a high cytotoxic activity against different cancer cell lines (Figure 5B). 

The high biological activity of meroterpenoids is probably associated with the addition of an isoprene fragment to the pharmacophore polyketide part that increases the affinity for biological membranes. The unique biological and structural properties of meroterpenoids contribute to the search for methods of their total and semi-synthetic synthesis [328,329,330]. 

Actinomycete-derived terpenoids participate in specific interactions with macroorganisms (plants and animals), regulate the bacterial life cycle, perform protective functions, or serve as taxonomic markers. Bacterial terpenoids are often optical isomers of plant terpenoids and may represent two chemical communication channels that do not overlap even if the same habitat is occupied by prokaryotic and eukaryotic organisms producing terpenes [103]. Soil-smelling terpenoids geosmin and 2-methylisoborneol were shown to play the role of signaling molecules for springtails (*Collembola*), which spread *Streptomyces* spores in the soil [331]. According to other reports, these terpenoids are aposematic signals used to indicate the unpleasant taste qualities of toxin-producing microbes, preventing predation by eukaryotes [332]. Čihák et al. (2017) pointed out that during germination of *S. coelicolor* M145 spores, they synthesize albaflavenone, which may coordinate the development of the producer (quorum sensing) and/or play a role in the competitive repression of microflora (quorum suppression) in the natural environment [117]. In the liquid culture, *S. coelicolor* A3(2) does not produce aminobacteriohopanetriol or produces this compound in negligible amounts. However, the triterpene generation increased sharply during the formation of an aerial mycelium and sporulation, which may be associated with structural changes in the membrane and protection against water loss [176]. In addition, some TSs and terpene derivatives are so unique that they can become a taxonomic trait and be used to identify different groups of actinomycetes. For instance, the bioinformatics analysis of all sequenced *Micromonospora* isolates revealed TS genes, which differ significantly from other groups of characterized bacterial TSs and may be useful as markers of the genus, while *Mycobacterium tuberculosis* H37Rvн produced specific diterpene nucleosides, 1- and *N^6^*-tuberculosinyladenosines, promising for development as specific diagnostic markers of tuberculosis.

Despite the significant (more than 300) number of publications on terpene biosynthesis by actinomycetes, the conducted patent analysis revealed only 26 patents in this research area (Table S1). Terpenoids such as linalool, geosmin, caryolan-1-ol, and pseudopterosin intermediates as well as meroterpenoids, namely napyradiomycins A4, A80915, bixiamycins, and sulfonylbixiamycins, were obtained from native or genetically modified streptomycetes, their genetic constructs, or individual terpene synthases. The relatively small number of active patents may be due to the initial stage of research in this area. In addition, wild-type strains are not suitable for commercial purposes, as they produce low quantities of target products.

**Table 2:** Biologically active terpene derivatives derived from actinomycetes.

Compound	Previously Isolated from Other Sources	Strain/Enzyme		Patent	Biological Activity	
**Mono- and sesquiterpenes**						
1,8-Cineole (**1**)	Yes	*Streptomyces clavuligerus* ATCC 27064	[53,54,55]	WO2018142109	anti-inflammatory antioxidant	[333]
Linalool (**2**)	Yes	*Streptomyces clavuligerus* ATCC 27064	[53,54,55]	WO2020234307 WO2018142109	anticancer antimicrobial neuroprotective anxiolytic antidepressant anti-stress hepatoprotective	[334]
		*Streptomyces* sp. GWS-BW-H5	[53]			
Nerolidol (**3**)	Yes	*Streptomyces clavuligerus* ATCC 27064	[53,54,55]	WO2018142109 WO2020234307	antimicrobial anti-biofilm antioxidant antiparasitic skin-penetration enhancer skin-repellent antinociceptive anti-inflammatory anticancer	[335]
α-Pinene (**7**) β-Pinene (**8**)	Yes	*Streptomyces coelicolor* A3(2)	[63]		antimicrobial	[336]
Limonene (**9**)	Yes	*Streptomyces coelicolor* A3(2)	[63]		antimicrobial antioxidant anti-inflammatory antidiabetic	[337]
γ-Terpinene (**10**) δ-Terpinene (**11**)	Yes	*Streptomyces coelicolor* A3(2)	[63]		antioxidant	[338]
(1*R*)-(+)-Camphor (**12**)	Yes	*Streptomyces coelicolor* A3(2)	[65]		insecticidal	[339]
(-)-*epi*-α-Bisabolol (**18**)	Yes	*Streptomyces citricolor* NBRC 13005	[67]		anti-inflammatory analgesic antibiotic anticancer	[340]
Germacrene B (**26**) Germacrene D (**24**)	Yes	TS from *Streptomyces pristinaespiralis* ATCC 25486	[82]		antileishmanial antiproliferative	[341]
		SAV76 from *Streptomyces avermitilis*	[83]			
		SpS from *Streptomyces xinghaiensis* S187	[84]			
		*Streptomyces hygroscopicus* NRRL 15879	[66]			
Bicyclogermacrene (**28**)	Yes	SpS from *Streptomyces xinghaiensis* S187	[84]		antibacterial antifungal	[342]
Isopterchiayione (**415**)	No	*Isoptericola chiayiensis* BCRC 16888	[262]		anti-inflammatory (IC_50_ 24.72 ± 1.25 µM)	[262]
Cyperusol C (**417**)	Yes	*Verrucosispora gifhornensis* YM28-088	[264]		antiviral (against hepatitis B virus, IC_50_ 14.1 ± 1.1 µM)	[343]
*epi*-Cubenol (**31**)	Yes	*Streptomyces* sp. GWS-BW-H5	[53]		antifungal	[344]
		Transf. *Streptomyces lividans* TK21 *gecA* from *Streptomyces griseus* IFO13350	[87]			
		*Streptomyces albolongus* YIM 101047	[73]			
		*Streptomyces griseus* NBRC102592	[88]			
		*Streptomyces roseosporus* NRRL 11379	[5]			
		*Streptomyces* sp. SirexAA-E	[5]			
		*Streptomyces roseosporus* NRRL15998	[237]			
		*Streptomyces flavogriseus* ATCC33331	[237]			
Kandenol A (**36**) Kandenol B (**37**) Kandenol C (**38**) Kandenol D (**39**) Kandenol E (**40**)	No	*Streptomyces* sp. HKI0595	[90]		antimicrobial (against *Bacillus subtilis*, *Mycobacterium vaccae*, MIC 12.5–50 µM)	[90]
(2*R*,4*S*,8α*R*)-8,8α,1,2,3,4-Hexahydro-2-hydroxy-4,8α-dimethyl-2(2H)-naphthalenone (**52**)	No	*Streptomyces* sp. XM17	[96]		antiviral (against influenza A virus, IC_50_ 5–49 nM)	[96]
(1*S*,3*S*,4*S*,4α*S*,8α*R*)-4,8α-Dimethyloctahydronaphthalene-1,3,4α(3H)-triol (**53**)						
(4*S*,4α*S*,8α*S*)-Octahydro-4α-hydroxy-4,8α-dimethyl-1(2H)-naphthalenone (**54**)						
(1β,4β,4aβ,8aα)-4,8α-Dimethyloctahydronaphthalene-1,4a(2H)-diol (**55**)	No	*Streptomyces albolongus* YIM 101047	[73]		antifungal (against *Candida parapsilosis*, MIC 3.13 µg/mL)	[73]
(-)-δ-Cadinene (**58**)	Yes	SSCG_02150 from *Streptomyces clavuligerus* ATCC 27074	[97]		antimicrobial	[345]
T-Muurolol (**59**)	Yes	SSCG_03688 from *Streptomyces clavuligerus* ATCC 27074	[97]		antifungal	[346]
		*Streptomyces* sp. M491	[98]			
15-Hydroxy-T-muurolol (**61**)	No	*Streptomyces* sp. M491	[98]		antitumor (IC_50_ 6.7 µg/mL)	[98]
10-*epi*-δ-Eudesmol (**86**)	Yes	*Streptomyces chartreusis* NRRL 3882	[5]		repellent (against *Aedes aegypti* and ticks)	[102,347]
β-Eudesmol (**72**)	Yes	*Streptomyces exfoliatus* SMF19	[66]		potential antitumor potential antiangiogenic antimicrobial	[348,349]
		*Streptomyces hygroscopicus* NRRL 15879	[66]			
Aromadendrene oxide-(2) (**79**)	Yes	*Streptomyces hygroscopicus* NRRL 15879	[66]		antibacterial antitumor	[350]
(-)-β-Cedrene (**126**) (+)-β-Cedrene (**127**)	Yes	*Streptomyces hygroscopicus* NRRL 15879	[66]	WO2015120431	antibacterial	[351]
		*epi*-isozizaene synthase from *Streptomyces coelicolor* A3(2)	[110,122]			
β-Patchoulene (**77**)	Yes	*Streptomyces hygroscopicus* NRRL 15879	[66]		anti-inflammatory	[352]
α-Elemol (**80**)	Yes	*Streptomyces parvulus* B1682	[66]		insecticidal (against *Ixodes scapularis, Amblyomma americanum*)	[353]
		*Streptomyces chartreusis* NRRL 3882	[102]			
Caryophyllene (**93**)	Yes	*Streptomyces yanglinensis* 3-10	[62]		anticancer antioxidant antimicrobial	[354,355]
		*Saccharothrix espanaensis* DSM 44229	[103]			
Caryolan-1-ol (**94**)	Yes	*Streptomyces griseus*	[105]		antifungal (against *Botrytis cinerea*, IC_50_ 0.026 µM/mL)	[107]
		Transf. *Streptomyces lividans* with *gcoA* from *S. griseus*				
		*Streptomyces globisporus* TFH56	[106]			
		*Streptomyces griseus* S4–7	[107]	WO2018062668		
		*Streptomyces albolongus* YIM 101047	[73]			
Albaflavenone (**109**)	No	*Streptomyces coelicolor* A3 (2)	[112]		antibacterial (against *Bacillus subtilis*, MIC 8–10 µg/mL)	[356]
		Transf. *Streptomyces avermitilis* SUKA16 with *sav3032* and *sav4925* from *S. avermitilis*	[119]			
		*Streptomyces cyaneogriseus* subsp. *noncyanogenus*	[5]			
		*Streptomyces spectabilis* NRRL-2792	[118]			
		*Streptomyces viridochromogenes* DSM 40736	[116]			
		*Streptomyces griseoflavus* Tu4000	[116]			
		*Streptomyces ghanaensis* ATCC 14672	[116]			
		*Streptomyces albus* ATCC 2396	[116]			
		*Streptomyces* sp. CRB46	[115]			
		*Streptomyces coelicolor* M145	[117]			
		*Streptomyces albidoflavus* DSM 5415		WO1995007878		
(Z)-α-Bisabolene (**115**) (Z)-γ-Bisabolene (**117**)	Yes	*epi*-isozizaene synthase *Streptomyces coelicolor* A3(2)	[110,122]	WO2015120431	antioxidant	[357]
Curcumene (**116**)	Yes	*epi*-isozizaene synthase *Streptomyces coelicolor* A3(2)	[110,122]		antifungal	[358]
Sesquiphellandrene (**118**)	Yes	*epi*-isozizaene synthase *Streptomyces coelicolor* A3(2)	[110,122]		antiproliferative	[359]
Strepsesquitriol (**136**)	No	*Streptomyces* sp. SCSIO 10355	[123]		anti-inflammatory	[123]
Pentalenolactone (**132**)	No	*Streptomyces exfoliatus* UC5319 *Streptomyces avermitilis* *Streptomyces arenae* TÜ469	[130]		antimicrobial antiviral	[125]
		*Streptomyces albus* JA 3453-10		DD261608		
1-Deoxy-8α-hydroxypentalenic acid (**150**)	No	*Streptomyces* sp. NRRL S-4	[134]		antimicrobial (against *Staphylococcus aureus*, MIC 16 μg/mL; *Escherichia coli*, MIC 16–32 μg/mL)	[134]
1-Deoxy-9β-hydroxy-11-oxopentalenic acid (**151**)						
Dihydro-β-agarofuran (**78**)	Yes	*Streptomyces hygroscopicus* NRRL 15879	[66]		insecticidal	[360]
Caryolan-1,9β-diol (**96**)	Yes	*Streptomyces* sp. AH25	[108]		anti-inflammatory (ED_50_ 0.34 mg/ear)	[361]
		*Streptomyces albolongus* YIM 101047	[73]			
Viridiflorol (**91**)	Yes	SAV_76 from *Streptomyces avermitilis*	[83]		anti-inflammatory antioxidant (against DPPH, IC_50_ 74.7 µg/mL)	[362]
**Di- and triterpenes and their derivatives**						
Lobocompactol (**166**)	No	*Streptomyces cinnabarinus* PK209	[140]		antifouling (against macroalga *Ulva pertusa*, EC_50_ 0.18 µg/mL; diatom *Navicula annexa*; EC_50_ 0.43 µg/mL)	[140]
Microeunicellol A (**168**)	No	*Streptomyces albogriseolus* SY67903	[142]		antitumor (against MCF-7, IC_50_ 5.3 μM; MDA-MB-231, IC_50_ 8.6 μM)	[142]
Terpentecin (**427**)	No	*Kitasatosporia griseola* MF730-N6	[202]		antibacterial (against *Staphylococcus aureus*, *Bacillus subtilis*, *Corynebacterium bovis*, *Shigella dysenteriae*, *Aeromonas salmonicida*, *Vibrio anguillarum*, MIC 0.05 µg/mL)	[274]
Isopimara-8(9),15-diene (**180**)	Yes	*Streptomyces* sp. PKU-TA00600	[150]		anti-inflammatory	[363]
		Sat1646 from *Salinispora* sp. PKU-MA00418				
Isopimara-7(8),15-diene (**445**) Isopimara-8(14),15-diene (**446**) *Syn*-isopimara-7(8),15-diene (**440**) 8β-Isopimara-9(11),15-diene (**441**) 8β-Pimara-9(11),15-diene (**442**) *Syn*-stemod-13(17)-ene (**443**) *Syn*-pimara-7(8),15-diene (**444**)	No					
2α-Hydroxy-8(14),15-pimaradien-17,18-dioic acid (**450**)	No	*Microbispora hainanensis* CSR-4	[281]		anti-Alzheimer neuroprotective (1 ng/mL) antitumor antioxidant	[281]
Gifhornenolone A (**447**)	No	*Verrucosispora gifhornensis* YM28-088	[264]		antiandrogenic (IC_50_ 2.8 µg/mL)	[264]
Actinomadurol (**452**)	No	*Actinomadura* sp. KC 191	[283]		antibacterial (against *Staphylococcus aureus*, *Kocuria rhizophila*, *Proteus hauseri*, MIC 0.39–0.78 μg/mL)	[283]
k4610422 (**453**)	No	*Actinomadura* sp. AMW41E2	[284]		cytotoxic (against P388, IC_50_ 30 μM)	[284]
Cyclooctatin (**184**)	No	*Streptomyces melanosporofaciens* MI614-43F2			anti-inflammatory	[364]
		Transf. *E. coli* with *CotB3* or *CotB4* from *Streptomyces afghaniensis*				
		*Streptomyces* sp. KCB17JA11				
3,7,18-Dolabellatriene (**188**)	Yes	Mutant W288G of CotB2 from *Streptomyces melanosporofaciens* MI614-43F2	[158]		antimicrobial (against methicillin-resistant *Staphylococcus aureus*, MIC 16.0 µg/mL)	[365]
2,7,18-Dolabellatriene (**459**)		*Saccharopolyspora spinosa* NRRL 18395	[286]			
Thunbergol (**464**)	Yes	*Allokutzneria albata* DSM 44149	[287]		antimicrobial	[366]
**Meroterpenoids**						
Furaquinocin A (**226**) Furaquinocin B (**227**)	No	*Streptomyces* sp. KO-3988 *Streptomyces* sp. CLl90	[185]	WO2006081537	antitumor (against HeLa S3, IC_50_ 1.6–3.1 μg/mL)	[185]
Furaquinocin C (**228**) Furaquinocin D (**226**) Furaquinocin E (**234**) Furaquinocin G (**235**) Furaquinocin H (**231**)	No	*Streptomyces* sp. KO-3988			cytotoxic (against B16, IC_50_ 0.08–6.87 μg/mL; HeLa S3, IC_50_ 0.22–5.05 μg/mL)	
Furaquinocin L (**238**)	No	*Streptomyces* sp. Je 1-369	[191]		antibacterial (against *Staphylococcus aureus*, MIC 2.0 μg/mL)	[191]
Murayaquinone (**240**)	No	*Streptomyces* sp. TBRC7642	[188]		antitubercular (MIC 3.13 μg/mL)	[188]
					cytotoxic (against MCF-7 IC_50_ 6.0 μM; NCI–H187, IC_50_0.85 μM; Vero, IC_50_2.05 μM)	
Merochlorin A (**241**)	No	*Streptomyces* sp. CNH-189	[192]		antibacterial (against MRSA, MIC 2.0–4.0 μg/mL; *Clostridium difficile* 0.3–0.15 μg/mL)	[192]
Merochlorin I (**249**)	No	*Streptomyces* sp. CNH-189	[194]		antibacterial (against *Bacillus subtilis*, MIC 1.0 μg/mL; *Kocuria rhizophila*, MIC 2.0 μg/mL; *Staphylococcus aureus*, MIC 2.0 μg/mL)	[194]
Merochlorin E (**245**) Merochlorin F (**246**)	No	*Streptomyces* sp. CNH-189	[193]		antibacterial (against *Bacillus subtilis,* MIC 1.0 µg/mL, *Kocuria rhizophila* MIC 2.0 μg/mL, *Staphylococcus aureus* MIC 1.0–2.0 μg/mL)	[193]
Flaviogeranin D (**256**) Flaviogeranin C2 (**258**)	No	*Streptomyces* sp. B9173	[196]		antibacterial (against *Mycobacterium smegmatis,* MIC 5.2 μg/mL)	[196]
					cytotoxic (against A549, IC_50_ 0.6–0.9 μM; Hela, IC_50_ 0.4–1.1 μM)	
Flaviogeranin A (**252**)		*Streptomyces* sp. RAC226	[195]		neuroprotective (EC_50_ 8.6 nM)	[195]
Naphterpin (**259**)	No	*Streptomyces* sp. CL190 *Streptomyces* sp. strain CLl90	[197]	WO2006081537	antioxidant (suppressed lipid peroxidation in rat homogenate system, IC_50_ 5.3 μg/mL)	[197]
Naphterpin B (**260**) Naphterpin C (**261**)	No	*Streptomyces* sp. CL190	[199]		antioxidant (suppressed lipid peroxidation in rat homogenate system, IC_50_ 6.0–6.5 μg/mL)	[199]
Napyradiomycin CNQ-525.1 (**226**)	No	*Streptomyces* sp. CNQ-525	[208]		antibacterial (against MRSA, MIC 1.95 μg/mL; *Enterococcus faecium* (VREF) MIC 1.9–3.9 μg/mL)	[208]
Napyradiomycin CNQ-525.2 (**281**)						
Napyradiomycin CNQ-525.3 (**282**)					cytotoxic (against HCT, IC_50_ 1.0–2.4 μg/mL)	
Napyradiomycin CNQ-525.4 (**283**)						
Napyradiomycin D1 (**287**)	No	*Streptomyces* sp. CA-271078	[203]		antibacterial (against MRSA, MIC 12.0–24.0 μg/mL; *Mycobacterium tuberculosis*, MIC 12.0–48.0 μg/mL)	[203]
					cytotoxic (HepG2, IC_50_ 14.9 μM)	
3-Dechloro-3-bromonapyradiomycin A1 (**266**)	No	*Streptomyces* sp. SCSIO 10428 *Streptomyces kebangsaanensis* WS-68302 *Streptomyces* sp. CA-271078	[201,204]	CN105399721	antibacterial (against *Staphylococcus aureus*, MIC 0.5–1.0 μg/mL; MRSA, MIC 4.0–8.0 μg/mL; *Bacillus subtilis*, MIC 1.0–2.0 μg/mL; *Bacillus thuringiensis*, MIC 0.5–2.0 μg/mL) cytotoxic (against HCT-116, IC_50_ 2.0–3.0 μM)	[201,204,209]
Napyradiomycin B1 (**273**)						
Naphthomevalin (**289**)						
Napyradiomycin A1 (**264**)	No	*Streptomyces* sp. CA-271078	[201]		antibacterial (against MRSA, MIC 0.5–1.0 μg/mL)	[201]
		*Streptomyces* sp. YP127	[200]		antiangiogenic	[200]
		*Streptomyces kebangsaanensis* WS-68302		CN105399721	antibacterial (against *Staphylococcus aureus*, MIC 0.078 µg/mL) antiviral (against *Pseudorabies virus*, IC_50_ 2.2 μg/mL)	
Napyradiomycin B2 (**275**)	No	*Streptomyces* sp. CNQ-329 *Streptomyces* sp. CNH-070	[206]		cytotoxic (against HCT-116, IC_50_ 3.18 μg/mL) antibacterial (against MRSA, MIC 3.0–6.0 μg/mL)	[206]
		*Streptomyces* sp. CA-271078	[203]			
Napyradiomycin B3 (**274**)	No	*Streptomyces* sp. CNQ-329 *Streptomyces* sp. CNH-070	[206]		cytotoxic (against HCT-116, IC_50_ 0.2 μg/mL) antibacterial (against MRSA, MIC 2.0 μg/mL; against *Staphylococcus aureus*, MIC 0.5 μg/mL; *Bacillus subtilis*, MIC 0.2 μg/mL; *Bacillus thuringiensis*, MIC 0.5 μg/mL)	[203,206]
		*Streptomyces* sp. SCSIO 10428	[203]			
Napyradiomycin B4 (**284**)		*Streptomyces* strains CNQ-329 and CNH-070	[206]		cytotoxic (against HCT-116, IC_50_ 1.41 μg/mL)	[206]
NPM 1 (**288**)		*Streptomyces* strains CNQ-329 and CNH-070	[206]		cytotoxic (against HCT-116, IC_50_ 4.2–4.8 μg/mL)	[206]
Napyradiomycin CNQ525.538 (**271**)	No	*Streptomyces* sp. CNQ-525	[209]		cytotoxic (against HCT-116, IC_50_ 6.0 μg/mL)	[209]
A80915A (**277**) A80915B (**278**) A80915D (**279**) A80915G (**291**)	No	*Streptomyces aculeolatus* A80915	-	EP0376609	antibacterial (against *Staphylococcus aureus*, MIC 0.03–4.0 μg/mL; *S. epidermidis,* MIC 0.15–2.0 μg/mL; *Streptococcus pyogenes*, MIC 0.03–2.0 μg/mL; *S. pneumonia*, MIC 0.125–2.0 μg/mL; *Enterococcus faecium,* MIC 1.0–4.0 μg/mL; *E. faecalis*, MIC 1.0 μg/mL; *Haemophilus influenzae*, MIC 0.008 μg/mL; *Clostridium difficile*, MIC 2.0–4.0 μg/mL; *C. perfringers*, MIC 2.0–4.0 μg/mL; *C. septicum*, MIC 1.0–2.0 μg/mL; *Eubacterium aerofaciens*, MIC 0.5–2.0 μg/mL; *Peptococcus asaccharolyticus*, MIC 0.5–4.0 μg/mL; *P. prevotii*, MIC 1.0–2.0 μg/mL; *P. intermediatus*, MIC 1.0–2.0 μg/mL; *Propionibacterium acnes*, MIC 0.5–1.0 μg/mL; *Bacteroides fragilis*, MIC 2.0–4.0; *B. melaninogenicus*, MIC 0.5–2.0 μg/mL; *B. corrodens*, MIC 2.0–4.0 μg/mL; *Fusobacterium symbiosum*, MIC 0.5–4.0 μg/mL)	-
A80915A (**277**) A80915B (**278**) A80915D (**279**)	No	*Streptomyces* sp. CNQ-525	[209]		cytotoxic (against HCT-116, IC_50_ 1.0–3.0 μg/mL)	[209]
7-Demethyl SF2415A3 (**272**) 7-Demethyl A80915B (**285**)	No	*Streptomyces antimycoticus* NT17	[202]		antibacterial (against *Staphylococcus aureus*, MIC 2.0–3.7 nM/mL; *Bacillus subtilis*, MIC 1.0–3.7 nM/mL)	[202]
Napyradiomycin A4 (**267**)	No	*Streptomyces kebangsaanensis* WS-68302		CN114805278	antiviral (against *Pseudorabies virus* (PRV), IC_50_ 2.056 μM)	
16*Z*-19-Hydroxynapyradiomycin A1 (**265**)	No	*Streptomyces* sp. YP127	[205]		anti-inflammatory antioxidant	[205]
(*R*)-3-Chloro-6-hydroxy-8-methoxy-alpha-lapachone (**286**)	No	*Streptomyces* sp. YP127 *Streptomyces antimycoticus* NT17	[202,205]		anti-inflammatory	[205]
Marfuraquinocin A (**292**) Marfuraquinocin C (**294**) Marfuraquinocin D (**295**)	No	*Streptomyces niveus* SCSIO 3406	[210]		cytotoxic (against NCI-H460, IC_50_ 3.7; 4.4; 8.8 μM) antibacterial (against *Staphylococcus aureus* ATCC 29213, methicillin-resistant *Staphylococcus epidermidis*, MIC 8.0 μg/mL)	[210]
FW03105 (**484**)	No	*Verrucosispora* sp. FIM06031		CN101921721	antitumor (against HepG2, IC_50_ 16.99 µM; EC109, IC_50_ 25.33 µM; HeLA, IC_50_ 34.64 µM)	
Saccharoquinoline (**492**)	No	*Saccharomonospora* sp. CNQ-490	[293]		cytotoxic (against HCT-116, IC_50_ 1.0 μM)	[293]
Teleocidin B (**314**)	No	*Streptomyces mediocidicus*	[211]		tumor promoter	[211]
		*Streptomyces* sp. 680560	[367]		nematicidal	[367]
		*Streptomyces blastmyceticus*	[214]			
Lavanducyanin (**304**)	No	*Streptomyces* sp. CNS-284 and CNY-960 *Streptomyces* sp. CLl90	[216]	WO2006081537	cytotoxic (against HCT-116, IC_50_ 2.41 μM)	[216]
					antimicrobial (against *Staphylococcus aureus*, MIC 2.92 μM; *Candida albicans*, MIC 5.96 μM)	
Marinocyanin A (**298**) Marinocyanin B (**299**) Marinocyanin C (**300**)	No	*Streptomyces* sp. CNS-284 и CNY-960	[216]	-	cytotoxic (against HCT-116, IC_50_ 0.029–0.049 μM)	[216]
					antimicrobial (against *Staphylococcus aureus*, MIC 2.37 μM; *Candida albicans*, MIC 0.95–3.90 μM)	
Farneside A (**306**)	No	*Streptomyces* sp. CNT-372	[217]		antimalarial (against *Plasmodium falciparum*)	[217]
Xiamycin A (**310**)		*Streptomyces* sp. SCSIO 02999	[220]	CN102757908 CN102732534	antiviral anti-HIV cytotoxic	[220]
		*Streptomyces* sp. GT2002/1503	[221]		antiviral (against SARS-CoV-2)	[368]
		*Streptomyces* sp. HKI0595	[226]		antiviral (against HSV-1)	[329]
Xiamycin methyl ester (**311**)	No	*Streptomyces* sp. SCSIO 02999	[220]	CN102757908	antitumor (IC_50_ 10.13 μM)	
					antiviral (against SARS-CoV-2)	[368]
Dixiamycin A (**328**) Dixiamycin B (**330**)	No	*Streptomyces* sp. GT2002/1503	[221]		antibacterial (against *E. coli*, *S. aureus*, MIC 8–16 µg/mL; *B. thuringiensis*, MIC 4–8 µg/mL)	[221]
		*Streptomyces xinghaiensis* NRRL B-24674T	[228]			
		*Streptomyces* sp. SCSIO 02999		CN102757908		
Dixiamycin 6a/6b (**333/334**)	No	Transf. *S. albus* with *xia* from *Streptomyces* sp. SCSIO 02999	[230]	WO2014029498	antibacterial (against MRSA, MIC 0.2 µg/mL)	[230]
Dixiamycin 8 (**337**)					antibacterial (against *S. aureus*, MRSA, MIC 1.56 µg/mL)	
Dixiamycin 7a/7b (**335**/**336**)	No	*Streptomyces olivaceus* OUCLQ19-3	[229]		antibacterial (*S. aureus*, *E. faecalis*, *E. faecium*, *M. luteus*, *P. aeruginosa*, MIC 6.25–12.5 µg/mL)	[229]
Dixiamycin 12a/12b (**331**/**332**)					antibacterial (*S. aureus*, MIC 0.78–3.12 µg/mL; *E. faecalis*, *E. faecium*, *M. luteus*, MIC 3.12–6.25 µg/mL; *P. aeruginosa*, MIC 1.56 µg/mL)	
Xiamycin B (**313**) Indosespene (**318**)	No	*Streptomyces* sp. HKI0595 *Streptomyces* sp. SCSIO 02999	[226]	CN102732534	antimicrobial (against MRSA; vancomycin-resistant *Enterococcus faecalis*)	[226]
Sespenine (**319**)					antiviral (against SARS-CoV-2)	[368]
Xiamycin D (**324**)	No	*Streptomyces* sp. HK18	[225]		antiviral (against PEDV)	[225]
Xiamycin C (**323**)					antiviral (against SARS-CoV-2)	[368]
Oridamycin A (**326**)	No	*Streptomyces* sp. KS84	[227]		antifungal (against *Saprolegnia parasitica*, MIC 3.0 µg/mL)	[227]
Sulfonylbixiamycin A (**338**)	No	Transf. *S. albus* with xiamycin BGC from *Streptomyces* sp.	[231]	WO2014029498	antibacterial (against *Bacillus subtilis*, MIC 6.25 µg/mL; *Staphylococcus aureus*, MIC 3.12 µg/mL; MRSA, MIC 6.25 µg/mL)	[231]
Cyslabdan A (**341**)	No	*Streptomyces cyslabdanicus* K04-0144	[233]		enhance (1000-fold) the antibiotic imipenem action (against MRSA)	[369]
Oxaloterpin A (**347**)	No	*Streptomyces* sp. KO-3988	[151]		antibacterial (against *Bacillus subtilis* ATCC 43223, IC_50_ 1.9 µM/mL; *Staphylococcus aureus* ATCC29213; EC_50_ 3.7)	[151]
		*Streptomyces griseus* CB00830	[235]			
		*Streptomyces* sp. SN194	[152]			
Chloroxaloterpin A (**352**) Chloroxaloterpin B (**353**)	No	*Streptomyces* sp. SN194	[152]		antifungal (against *Botrytis cinerea*, EC_50_ 4.40–4.96 µg/mL)	[152]
Fusicomycin A (**384**) Fusicomycin (**385**) Fusicomycin B (**386**)	No	*Streptomyces violascens* YIM 100212	[164]		cytotoxicity (against BGC-823 H460, HCT116, HeLa, SMMC7721 8.9, IC_50_ from 3.5 ± 0.7 to 14.1 ± 0.8 µM)	[164]
Streptooctatin A (**387**) Streptooctatin B (**388**)	No	*Streptomyces* sp. KCB17JA11	[243]		autophagic (against HeLa)	[243]
Actinoranone (**389**)		*Streptomyces* sp. CNQ-027	[244]		cytotoxic (against HCT-116, LD_50_ 2.0 μg/mL)	[244]
Brasilicardin A (**490**)	No	*Nocardia brasiliensis* IFM 0406 (now *N. terpenica*)	[299]		immunosuppressive	[300]
					antiproliferative (against LN229, IC_50_ 0.13 μM)	[306]
Platensimycin (**390**) Platencin (**391**)		*Streptomyces platensis* MA7327 *Streptomyces platensis* MA7339 *Streptomyces platensis* MA7237	[245,246]	US20090081673	antibacterial (against *S. aureus* (MRSA), *Enterococcus faecalis*, *Enterococcus faecium*, MIC 0.1–1.0 μg/mL)	[245,246]
Atolypene A (**497**) Atolypene B (**498**)	No	Transf. *Streptomyces albus* with *ato* gene cluster from *Amycolatopsis tolypomycina* NRRL B-24205	[307]		cytotoxic (against HL-60, Jurkat, HEK293, HeLa, A549, IC_50_ 12.0–36.7 μM)	[307]
Terretonin N (**499**)	No	*Nocardiopsis* sp. LGO5	[308]		antibacterial (against *Staphylococcus warneri*)	[308]
Soyasaponin I (**407**)	Yes	*Streptomyces* sp. YIM 56130	[94]		anti-inflammatory antimutagenic anticarcinogenic antimicrobial	[256]
Longestin (**408**)	No	*Streptomyces argenteolus* A-2	[257]		antiamnesic (IC_50_ 0.065 µM)	[370]

## 4. Conclusions

Thus, the synthesis of terpenes and terpenoids is an important pathway in the secondary metabolism of actinomycetes. The compounds produced may be promising therapeutic agents for the treatment of viral, inflammatory, cancerous, and other diseases in the future. Terpenoids and meroterpenoids synthesized by actinomycetes and possessing high antibacterial activity against drug-resistant pathogenic microorganisms may be useful for the development of new antibiotics. Further study of actinomycetes, accumulation of genetic information about this group of microorganisms, and employment of modern and development of novel tools of synthetic biology and genetic engineering will open prospects for creation of ideal “cell factories” using actinomycetes. 

## Figures and Tables

**Table 1:** Potential applications of secondary metabolites produced by actinomycetes in various fields of human activities.

Application Area		Review, Book Chapter
Agriculture	Plant growth promoting	[27]
	Phytopathogen control	[28]
	Bioherbicides	[20]
	Biopesticides	[29]
Bioinsecticides	Against insects, mites	[30]
Medicine	Antibiotics	[26,31,32]
	Pharmaceuticals (antitumor, anti-inflammatory, antifungals, antihelminthics, etc.)	[33,34,35,36,37]
	Probiotics	[38,39]
Industry	Detergents (Surfactants)	[40]
	Biofuel	[8]

## Data Availability

Data sharing not applicable.

## Supplementary Materials

The following supporting information can be downloaded at: https://www.mdpi.com/article/10.3390/ph16060872/s1. Table S1: Patents on the biosynthesis of terpene derivatives using actinomycetes.
