# Supplementary material for: Actinomycetes as Producers of Biologically Active Terpenoids: Current Trends and Patents
Source: Pharmaceuticals (Basel). 2023 Jun 12;16(6):872. doi: 10.3390/ph16060872 (PMC10301674; doi:10.3390/ph16060872)
Supplement: Supplementary file 1 [file pharmaceuticals-16-00872-s001.zip › pharmaceuticals-2314817-supplementary.pdf]

Table S1. Patents on the biosynthesis of terpene derivatives using actinomycetes.

	Patent	Year	Country	Title	Assignee/applicant	Inventors	Strains/enzymes
1	WO2022003167<br>EP3933038	2022<br>2022	-<br>European Patent Office	MODIFIED TERPENE SYNTHASES AND THEIR USE FOR PRODUCTION OF PSEUDOPTEROSIN INTERMEDIATES AND/OR PSEUDOPTEROSINS	Technische Universität München	Ringel, Marion Brück Reinbold, Thomas Garbe, Markus Daniel	Modified hydropyrene synthase from *Streptomyces clavuligerus* ATCC 27064
2	CN102719376	2012	China	STRAIN FOR PRODUCING GEOSMIN	Jiangnan University	Xu, Yan<br>Fan, Wenlai<br>Du, Hai	*Streptomyces albus* LBG-FXJ (CGMCC 4206)
3	CN102181392	2011	China	STRAIN FOR GENERATING GEOSMIN AND SCREENING METHOD THEREOF			*Streptomyces fradiae* FJ-HX (CGMCC 4205)<br>*Streptomyces* sp. QC-1 (CGMCC 4535)<br>*Streptomyces* sp. QC-2 (CGMCC 4536)
4	CN102719375	2012	China	GEOSMIN PRODUCING STRAIN			*Streptomyces* sp. QC-1 (CGMCC 4535)
5	CN102719377	2012	China	GEOSMIN PRODUCING STRAIN			*Streptomyces* sp. QC-2 (CGMCC 4536)
6	WO2018062668<br>KR1018164420000<br>US20200015478	2018<br>2018<br>2020	-<br>Republic of Korea<br>United States of America	ANTIBACTERIAL COMPOSITION CONTAINING CARYOLAN-1-OL	Industry-Academic Cooperation Foundation Gyeongsang National University	Kwak, Youn-Sig Park Cho, Jung-Kyu Gyeong-Jun Kim, Jun-Heon	*Streptomyces* sp. S4–7
7	WO2018142109<br>CN110268065<br>EP3577226<br>IN201917029931<br>US20200102585	2018<br>2019<br>2019<br>2019<br>2020	-<br>China<br>European Patent Office<br>India<br>United States of America	PROCESS OF PRODUCING MONOTERPENES	C3 Bio-Technologies Limited	Karuppiah, Vijaykumar G. H. Leferink, Nicole S. Scrutton, Nigel	*Streptomyces clavuligerus* ATCC 27064

8	US20210238640	2021	United States of America	PROCESS OF PRODUCING MONOTERPENES	C3 Bio-Technologies Limited	Karuppiah, Vijaykumar<br>G. H. Leferink, Nicole<br>S. Scrutton, Nigel	Monoterpene synthases from *Streptomyces clavuligerus* ATCC 27064
9	WO2020234307<br>EP3824081	2020<br>2021	-<br>European Patent Office	LINALOOL SYNTHASES	C3 Bio-Technologies Limited	Scrutton, Nigel<br>Leferink, Nicole	Linalool synthases from *Streptomyces clavuligerus*
10	US20200239796	2020	United States of America	HOST CELLS AND METHODS FOR PRODUCING TRICYCLIC SESQUITERPENES, AVIATION AND MISSILE FUEL PRECURSORS	The Regents of the University of California	Taek, Soon Lee	*Streptomyces* sp. UC5319<br>*Streptomyces coelicolor* A3(2)
11	WO2015120431	2015	-	NOVEL TERPENE CYCLASE VARIANTS, AND METHODS USING SAME	The Trustees Of The University Of Pennsylvania<br>Brown University	Christianson, David W.<br>Li, Ruiqiong<br>Litwin, Kevin<br>Cane, David E.<br>Chou, Wayne<br>Harris, Golda<br>Himmelberge, Julie Anne	*Epi*-isozizaene synthase from *S. coelicolor* A3(2)
12	JP2019149945	2019	Japan	METHOD FOR PRODUCING PLATENSIMYCIN	Univ Of Tokyo Toyota Boshoku Corp	Onaka, Hiroyasu Asamizu, Shumpei Kawai, Sung-Jin	*Streptomyces hygroscopicus* HOK021 (NITE P-02560)
13	US20090081673	2009	United States of America	PLATENSIMYCIN BIOSYNTHETIC GENE CLUSTER OF STREPTOMYCES PLATENSIS	Wisconsin Alumni Research Foundation<br>Shen Ben<br>Smanski Michael J.	Smanski, Michael J.<br>Shen, Ben	*Streptomyces platensis* SB12002, SB12600
14	CN101921721	2010	China	NEW MARINE VERRUCOSISPORA SP.FIM06031 AND APPLICATION THEREOF	Fujian Institute of Microbiology	Jiang, Hong<br>Nie, Yilei<br>Lin, Ru<br>Lian, Yunyang<br>Zheng, Wei	*Verrucosispora* sp. FIM06031
15	CN101898936	2010	China	NOVEL ANTITUMOR TERPENOID FW03105	Fujian Institute of Microbiology	Nie, Yilei<br>Jiang, Hong<br>Lin, Ru	*Verrucosispora* sp. FIM06031

						Zheng, Yongbiao<br>Xu, Liyan<br>Lian, Yunyang<br>Zheng, Wei	
16	WO2015022798<br>EP3034610<br>JPWO2015022798	2015<br>2016<br>2015	-<br>European Patent Office<br>Japan	NOVEL TERPENOID COMPOUND AND METHOD FOR PRODUCING SAME	Kitasato Inst<br>Nagase & Co Ltd	Ikeda, Haruo<br>Sota, Masahiro	Mutants of *Streptomyces avermitilis* ATCC 31267
17	WO2015200501	2015	-	STRAIN PRIORITIZATION FOR NATURAL PRODUCT DISCOVERY BY A HIGH THROUGHPUT REAL-TIME PCR METHOD	The Scripps Research Institute	Shen, Ben	*Streptomyces platensis* spp.
18	DD261608	1988	Germany	METHOD OF PREPARING A SESQUITERPEN ANTIBIOTIC	ADW DDR	Fleck, Werner<br>Graefe, Udo<br>Reinhardt, Guenter<br>Schade, Wolfgang<br>Tresselt, Dieter-Klaus	*Streptomyces albus* JA 3453-10
19	EP2885274<br>WO2014029498	2015<br>2014	European Patent Office<br>-	BIXIAMYCINS AND SULFONYLBIXIAMYCINS	Leibniz Institute Fuer Naturstoff Forschung Und Infektionsbiologie Hans Knoell Institute	Hertweck, Christian<br>Ding, Ling<br>Xu, Zhongli<br>Baunach, Martin	Transf. *Streptomyces albus* with *xia* from *Streptomyces* sp. SCSIO 02999
20	CN102732534	2012	China	BIOSYNTHETIC GENE CLUSTER OF XIAMYCIN A AND OXIAMYCIN, AND APPLICATION THEREOF	South China Sea Institute of Oceanology, Chinese Academy of Sciences	Zhang, Changsheng<br>Li, Huixian<br>Zhang, Qingbo<br>Li, Sumei<br>Zhu, Yiguang<br>Zhang, Guangtao<br>Zhang, Haibo<br>Tian, Xinpeng<br>Zhang, Si<br>Ju, Jianhua	*Streptomyces* sp. SCSIO 02999
21	CN102757908	2012	China	STREPTOMYCES SP., INDOLE SESQUITERPENOIDS, AND	South China Sea Institute of	Zhang, Changsheng<br>Zhang, Qingbo	*Streptomyces* sp. SCSIO 02999

				PREPARATION METHOD AND APPLICATION OF INDOLE SESQUITERPENOIDS TO PREPARATION OF ANTIBACTERIAL AND ANTITUMOR MEDICAMENT	Oceanology, Chinese Academy of Sciences	Li, Sumei<br>Tian, Xinpeng<br>Zhang, Wenjun<br>Zhang, Haibo<br>Li, Huixian<br>Zhang, Si<br>Ju, Jianhua	
22	CN114805278	2022	China	NAPYRADIOMYCIN A4 AND APPLICATION THEREOF IN RESISTING PORCINE PSEUDORABIES VIRUS	Hubei Biopesticide Engineering Research Center	Zhang, Yani<br>Fang, Wei<br>Liu, Manli<br>Wang, Kaimei<br>Wan, Zhongyi<br>Zhang, Zhigang<br>Liu, Fang<br>Wu, Zhaoyuan<br>Ke, Shaoyong<br>Shi, Liqiao	*Streptomyces kebangsaanensis* WS-68302
23	CN105399721	2016	China	NEW COMPOUNDS AND PREPARATION METHOD THEREOF, AND APPLICATIONS OF NEW COMPOUNDS IN PREPARATION OF ANTIBACTERIAL AND ANTITUMOR DRUGS	South China Sea Institute of Oceanology, Chinese Academy of Sciences	Zhang, Changsheng<br>Wu, Zhengchao<br>Li, Sumei<br>Li, Jie<br>Kumar, Saulaf<br>Zhang, Qingbo<br>Zhang, Haibo<br>Zhang, Wenjun<br>Si, Zhang	*Streptomyces* sp. SCSIO 10428
24	JP1988051395	1988	Japan	ANTIBIOTIC SF-2415 AND PRODUCTION THEREOF	Meiji Seika Co., Ltd.	Ouchi, Shokichi<br>Gomi, Shuichi<br>Watanabe, Hiroomi<br>Nakazawa, Tadashi<br>Shomura, Takashi<br>Sezaki, Masatsugu	*Streptomyces aculeolatus* SF 2415
25	WO2006081537	2006	-			Kuzuyama, Tomohisa	*Streptomyces* sp. CLl 90

	US20100285502	2010	United States of America	WO2006081537 - NOVEL AROMATIC PRENYLTRANSFERASES, NUCLEIC ACIDS ENCODING SAME AND USES THEREFOR	The Salk Institute For Biological Studies, Toudai Tlo, Ltd	Noel, Joseph, P.<br>Richard, Stephane, P.	
	US20080274478	2008	United States of America				
	KR1020070101359	2007	Republic of Korea				
	JP2008528036	2008	Japan				
	US20060183211	2006	United States of America				
	CN101137663	2006	China				
26	CA2006342	1990	Canada	ANTIBIOTIC A80915 AND PROCESS FOR ITS PRODUCTION	-	Fukuda, David Shuichi<br>Mynderse, Jon Stuart<br>Yao, Raymond Che-Fong	*Streptomyces aculeolatus* NRRL 18422
	JP1990258774	1990	Japan				
	AT111092	1994	Austria				
	DE000068918071	1995	Germany				
	US4904590	1990	United States of America				
	ES2059791	1994	Spain				
	EP0376609	1990	European Patent Office				
